# Effectiveness of Wrist-Worn Wearable–Guided Cardiac Rehabilitation Delivered Through Fully Remote or Hybrid Programs in Patients With Cardiovascular Disease: Systematic Review and Meta-Analysis of Randomized Controlled Trials

**DOI:** 10.2196/101306

**Published:** 2026-10-27

**Authors:** Jun Hwan Choi, So Young Lee, Kyung Cheon Seo, Sang Yoon Lee, Hyo Kyung Lee, Dongbin Heo, Hyun Ryu, Jong Hoon Kim, Jeong Eul Kwon, Bo Ryun Kim, Michael Y Lee

**Affiliations:** 1 Department of Rehabilitation Medicine Jeju National University Hospital Jeju National University Jeju Republic of Korea; 2 Department of Physical Medicine and Rehabilitation Korea University Anam Hospital Korea University College of Medicine Seoul Republic of Korea; 3 Department of Rehabilitation Medicine Seoul National University Hospital Seoul National University Seoul Republic of Korea; 4 School of Industrial Management Engineering Korea University College of Engineering Seoul Republic of Korea; 5 H Ben Taub Department of Physical Medicine and Rehabilitation Baylor College of Medicine Houston, TX United States

**Keywords:** cardiac rehabilitation, cardiovascular diseases, digital health, randomized controlled trials as topic, wearable electronic devices

## Abstract

**Background:**

Cardiovascular disease (CVD) remains the leading cause of death worldwide, yet participation in cardiac rehabilitation (CR) remains suboptimal. Wrist-worn wearables may support fully remote or hybrid CR through activity monitoring and individualized feedback, but the effectiveness and consistency of benefits remain uncertain.

**Objective:**

This study aimed to evaluate the effectiveness of wrist-worn wearable–guided CR delivered through fully remote or hybrid programs initiated during phase II or III CR in patients with CVD.

**Methods:**

A total of 8 databases were searched for randomized controlled trials published from January 1, 2015, through August 7, 2026. Eligible trials enrolled adults with CVD and integrated a wrist-worn wearable into fully remote or hybrid CR. Studies focused primarily on heart failure or using wearables only for outcome measurement were excluded. Primary outcomes were peak oxygen consumption (VO_2_), 6-minute walk distance (6MWD), and daily steps. Random-effects meta-analyses used mean differences (MDs), except for quality of life, which used standardized MDs. Between-study variance was estimated using the DerSimonian-Laird method, pooled-effect CIs used the Knapp-Hartung adjustment, and 95% prediction intervals (PIs) used the Nagashima-Noma-Furukawa parametric bootstrap method. Risk of bias was assessed with Cochrane Risk of Bias 2. Subgroup and univariable meta-regression analyses explored study and intervention characteristics.

**Results:**

A total of 18 trials involving 1993 randomized participants, reported in 20 publications, were included; 17 contributed to meta-analysis. Pooled estimates favored wearable-guided CR for peak VO_2_ (10 studies; 591 participants; MD 1.69, 95% CI 0.46-2.92, 95% PI –2.21 to 5.39 mL/kg/min), 6MWD (9 studies; 1094 participants; MD 31.03, 95% CI 7.66-53.92, 95% PI –62.11 to 123.18 m), and daily steps (8 studies; 474 participants; MD 1062.8, 95% CI 383.5-1825.0, 95% PI –503.0 to 2860.0 steps/day). All 3 PIs included the null, indicating uncertainty about consistency across future comparable settings. No clear pooled effects were observed for anaerobic threshold, BMI, waist circumference, grip strength, or quality of life. Subgroup differences were not statistically significant. In univariable meta-regression, a higher proportion of female participants was associated with greater peak VO_2_ improvement, although this aggregate-level finding was exploratory.

**Conclusions:**

Wrist-worn wearable–guided CR delivered through fully remote or hybrid programs may improve average exercise capacity and physical activity in patients with CVD. However, PIs included the null, indicating uncertainty about consistency across future settings. These programs may extend activity monitoring, individualized feedback, and behavioral support beyond center-based CR, but their multicomponent nature prevents attributing effects to wearables alone. Larger, rigorously designed trials with standardized protocols and outcomes should identify the patients and program components most likely to benefit. Future research should also evaluate long-term adherence, safety, equity, and implementation within routine multidisciplinary CR across diverse health systems.

## Introduction

Cardiovascular disease (CVD) remains the leading cause of death worldwide. Annual deaths are projected to rise from 20.5 million in 2025 to 35.6 million in 2050, with global prevalence increasing by approximately 90% [1]. Its economic burden is also substantial; in the United States, annual cardiovascular health care costs are projected to reach US $1.49 trillion by 2050, with an additional US $361 billion in productivity losses [2]. Long-term risk reduction and functional recovery therefore remain major public health priorities.

Cardiac rehabilitation (CR) is a core component of secondary prevention, integrating individualized exercise, risk-factor management, education, and psychosocial support [3]. It reduces cardiovascular mortality and readmissions and improves exercise capacity and quality of life (QoL) [4,5], yet participation and completion remain suboptimal [6,7]. Travel, limited program availability, work and caregiving responsibilities, socioeconomic constraints, and physical limitations restrict engagement [8-10]. Home-based, community-based, remote, hybrid, and digitally supported models have therefore been developed to extend monitoring and behavioral support beyond centers [11-15].

Wearable devices are body-worn sensors that collect and transmit physiological or behavioral data, including chest-strap monitors, biosensors, smart garments, smartwatches, and activity trackers [15]. Previous reviews examined wearable activity monitoring during CR maintenance, heterogeneous wearable-assisted home CR, broader eHealth interventions, or mixed devices including pedometers and accelerometers [16-19]. They did not isolate actively guided wrist-worn programs initiated during phase II or III CR. Wrist-worn devices are clinically relevant because they are accessible, scalable, and can continuously monitor heart rate (HR) and activity with low user burden. Several recent randomized controlled trials (RCTs) also warrant updated synthesis.

Individual RCTs of wrist-worn wearable–guided CR have been small and inconsistent: Zhang et al [20] and Zheng et al [21] reported significant peak oxygen consumption (VO_2_) gains, whereas Yagi et al [22] and Mitropoulos et al [23] did not. Effects on exercise capacity, physical activity, and related outcomes therefore remain uncertain.

This systematic review and meta-analysis evaluated wrist-worn wearable–guided CR delivered through fully remote or hybrid programs initiated during phase II or III CR in patients with CVD. We assessed exercise capacity, physical activity, and related outcomes and explored heterogeneity through subgroup and meta-regression analyses of participant, intervention, and comparator characteristics.

## Methods

This systematic review and meta-analysis of RCTs followed PRISMA (Preferred Reporting Items for Systematic Reviews and Meta-Analyses) 2020 [24]. The completed expanded checklist is provided in Multimedia Appendix 1.

### Eligibility Criteria

Eligibility was defined using population, intervention, comparator, outcomes, and study design framework. We included adults with CVD receiving wrist-worn wearable–guided CR delivered through fully remote or hybrid programs initiated during phase II or III. Phase denoted when the intervention began, not eligibility of all delivery settings. Both phases were included to span structured postacute rehabilitation through longer-term maintenance; phase was extracted as a prespecified subgroup variable. Intervention duration was unrestricted.

Eligible participants included patients with coronary artery disease, acute coronary syndrome, ischemic heart disease, and those who had undergone percutaneous coronary intervention (PCI) or cardiac surgery, such as coronary artery bypass grafting, valve surgery, or aortic surgery, or transcatheter valve intervention. Studies primarily focused on patients with heart failure were excluded; however, studies enrolling mixed populations of patients with cardiovascular disease remained eligible when patients with heart failure constituted only a subset of the overall population.

Eligible interventions used a wrist-worn smartwatch or smartband as a core component of structured remote or hybrid CR to guide exercise prescription, monitor physical activity, provide individualized feedback, or manage adherence. Conventional center-based CR alone and wearable use solely for passive monitoring or outcome assessment were ineligible. Comparators included usual care, unsupported home exercise, passive or minimally supported monitoring, and structured center-based CR.

Eligible studies were full-text RCTs published in English or Korean from January 1, 2015, through August 7, 2026. Conference abstracts, study protocols, review articles, nonrandomized or observational studies, and secondary or overlapping reports without independent eligible outcome data were excluded. Gray literature, including conference abstracts, dissertations, and unpublished materials, was not included because the review was restricted to full-text, peer-reviewed RCTs providing sufficient methodological information for eligibility and risk-of-bias assessment.

### Information Sources

We searched PubMed, Web of Science, CENTRAL, Embase, Scopus, CINAHL, KoreaMed, and KMbase. The original search (November 14, 2025) was updated on August 7, 2026, covering January 1, 2015, through August 7, 2026. Reference lists of included reports and relevant reviews were examined. Trial registries, regulatory databases, and gray literature were not searched.

### Search Strategy

Following PRISMA-S (Preferred Reporting Items for Systematic Reviews and Meta-Analyses Literature Search Extension) [25], database-specific strategies combined controlled vocabulary and free-text terms for CVD or cardiac procedures, wrist-worn wearables or remote monitoring, CR or related delivery models, and RCTs. Syntax, field tags, proximity operators, and filters were adapted by database. The August 7, 2026, update used the same concepts and updated date limits; overlapping records were deduplicated when day-specific restrictions were unavailable.

Complete original and updated strategies, platforms, dates, limits, filters, and yields are provided in Multimedia Appendix 2. No automated search tools were used; the strategies did not undergo peer review using the Peer Review of Electronic Search Strategies, and authors or manufacturers were not contacted.

### Selection Process

Records were exported to EndNote (version 21; Clarivate) and deduplicated. Two reviewers (BRK and JHC) independently screened titles and abstracts; reports considered potentially eligible by either were retrieved. Four reviewers (BRK, JHC, S Young Lee, and KCS) independently assessed and cross-checked full texts, recorded exclusion reasons, and resolved disagreements by consensus. No automation or crowdsourcing was used, and no translation was required.

### Data Collection Process

Three reviewers (BRK, JHC, and S Young Lee) extracted data using a standardized Excel form; 4 reviewers cross-checked entries and resolved discrepancies by consensus. Extracted variables covered study design; participant characteristics; CR phase and delivery model; intervention, wearable, feedback, exercise, and comparator characteristics; outcomes; and assessment timing.

Companion reports were linked as one study, with outcome data taken from the relevant report. Each cohort contributed only 1 estimate per outcome, preventing double counting. No automated extraction tools were used.

### Data Items

Primary outcomes were peak VO_2_, 6-minute walk distance (6MWD), and daily steps. Peak VO_2_ was the highest directly measured oxygen uptake during maximal or symptom-limited cardiopulmonary exercise testing; relative values (mL/kg/min) were preferred, and equivalent VO_2_ max measures were included. The 6MWD represented submaximal functional capacity, and daily steps represented habitual ambulatory activity rather than functional independence in activities of daily living.

Secondary outcomes included anaerobic threshold (AT), BMI, waist circumference, grip strength, and QoL. Peak VO_2_ and AT were expressed in mL/kg/min, 6MWD in meters, daily steps in steps/day, BMI in kg/m^2^, waist circumference in centimeters, and grip strength in kilograms. QoL was assessed using validated instruments, including the 36-Item Short Form Health Survey (SF-36), 12-Item Short Form Health Survey, Kansas City Cardiomyopathy Questionnaire, World Health Organization Quality of Life-Brief version, and EQ-5D. Additional outcomes reported by the included studies were recorded descriptively but were not included in the predefined quantitative syntheses.

We also extracted study setting and design; diagnosis; randomized, analyzed, and outcome-specific sample sizes; dropout; age; sex; intervention duration and dose; follow-up; and comparator components. Combined exercise included aerobic and resistance training; comparators were categorized as minimal/usual care, passive or less intensive wearable monitoring, or active structured CR.

Information that was missing or unclear in the published reports was recorded as not reported and was not imputed unless it could be directly calculated from the available data. When only the proportion of male participants was reported, the proportion of female participants was calculated as 100% minus the reported male proportion. Randomized, analyzed, and outcome-specific sample sizes were extracted separately when available; the sample size used in each meta-analysis was determined according to the population contributing to the corresponding outcome estimate, as described in the Synthesis Methods section.

### Study Risk of Bias Assessment

Risk of bias was assessed at the outcome level using the revised Cochrane Risk of Bias 2 (RoB 2) tools in accordance with the Cochrane Handbook for Systematic Reviews of Interventions [26,27]. Four reviewers (BRK, JHC, S Young Lee, and KCS) independently assessed the studies assigned to them, with each outcome-specific assessment performed by at least 2 reviewers. The reviewers subsequently discussed their assessments, and disagreements were resolved by consensus.

The standard RoB 2 tool was used for parallel RCTs, the cluster-specific tool for the cluster RCT, and the crossover tool for the crossover RCT. Design-specific judgments, including recruitment timing and period or carryover effects, were mapped to harmonized D1-D5 labels for Multimedia Appendix 3.

Domain and overall judgments followed RoB 2 algorithms: low risk required all domains to be low; some concerns required at least one concern without a high-risk domain; and high risk reflected at least one high-risk domain or multiple concerns substantially lowering confidence. Plots used R (version 4.5.2; R Foundation for Statistical Computing) [28]; no automation was used.

### Effect Measures

All outcomes were continuous. Mean differences (MDs) with 95% CIs were used when outcomes were reported using a common measurement scale. Accordingly, MDs were calculated for peak VO_2_ and AT in mL/kg/min, 6MWD in meters, daily steps in steps/day, BMI in kg/m^2^, waist circumference in centimeters, and grip strength in kilograms. For QoL, which was assessed using different validated instruments across studies, standardized mean differences (SMDs; Hedges *g*) with 95% CIs were calculated. Before calculating SMDs, the direction of each QoL scale was harmonized so that effect estimates had a consistent interpretation.

Positive MDs represented effects favoring the intervention for peak VO_2_, AT, 6MWD, daily steps, and grip strength, whereas negative MDs represented effects favoring the intervention for BMI and waist circumference. For QoL, the direction of effect was defined consistently after harmonization of the scales. No prespecified thresholds were used to categorize pooled effects as small, moderate, or large.

### Synthesis Methods

Outcome-specific meta-analyses included studies with clinically comparable measures and sufficient data to calculate an effect and variance. Otherwise, studies remained in the review and risk-of-bias assessment but not the synthesis. Postintervention values were preferred; change scores were used when unavailable. Units were converted as needed.

For studies reporting outcomes at multiple follow-up time points, only 1 assessment per outcome was included to avoid repeated inclusion of the same participants. The assessment conducted at or closest to the completion of the intervention was preferentially selected. When an assessment at intervention completion was unavailable, the earliest eligible postintervention assessment was used. For each outcome, the sample size corresponding to the reported summary statistics was used. The randomized sample size was used when results were reported according to the intention-to-treat principle, whereas the outcome-specific analyzed sample size was used when analyses were based on participants with available outcome data. Missing outcome data were not imputed unless the required statistics could be derived directly from information reported in the original article using established meta-analytic methods.

Multiple reports originating from the same trial cohort were linked and treated as companion publications. When companion reports provided data for different outcomes, data were extracted from the report relevant to each outcome. Each trial cohort contributed only one effect estimate to any given outcome-specific meta-analysis. For multiarm trials, only the comparison relevant to the eligibility criteria was included, and no shared intervention or comparator group was entered more than once in the same pooled analysis. Thus, no participant was double-counted within any outcome-specific meta-analysis.

MDs with 95% CIs were calculated for peak VO_2_, 6MWD, daily steps, AT, BMI, waist circumference, and grip strength because these outcomes were reported using consistent measurement units. For QoL, SMDs using Hedges *g* with 95% CIs were calculated because different validated instruments were used across studies. Quantitative synthesis was restricted to validated overall or composite QoL scores with sufficient numerical data to calculate an effect estimate and variance. An unvalidated sum of SF-36 domain scores was not considered an eligible overall QoL score. Where necessary, score directions were harmonized so that higher values consistently represented better QoL.

Pooled effect estimates were calculated using inverse-variance random-effects models because clinical and methodological heterogeneity was anticipated across study populations and interventions. Between-study variance (τ^2^) was estimated using the DerSimonian-Laird method, and 95% CIs for pooled effects were calculated using the Knapp-Hartung adjustment. Statistical heterogeneity was described using Cochran Q, τ^2^, and *I*^2^. *I*^2^ was not interpreted using fixed low, moderate, or high categories because it represents the relative proportion of observed variability attributable to between-study heterogeneity rather than its absolute magnitude or clinical importance and can be imprecise when few studies are available. Heterogeneity was therefore interpreted jointly using τ^2^, 95% prediction intervals, and clinical and methodological differences among studies [29,30].

For the main meta-analyses, 95% prediction intervals were calculated using the Nagashima-Noma-Furukawa parametric bootstrap method [31] to estimate the range in which the effect of a future comparable study might be expected. Prediction intervals were not calculated for subgroup analyses because of the small number of studies within individual subgroups and the resulting instability of subgroup-specific estimates. A reliable 95% prediction interval was not reported for QoL because marked between-study variability remained after exclusion of the study with an invalid SF-36 total score. Forest plots presented study-specific estimates, Knapp-Hartung-adjusted pooled estimates, and clearly labeled normal-normal framework (NNF) prediction intervals.

Subgroup and meta-regression analyses were planned to investigate potential sources of heterogeneity and patient- and intervention-level factors associated with intervention effectiveness. The specific outcomes eligible for these analyses were determined after data extraction based on the number of contributing studies and the availability of relevant subgroup and study-level covariate data, rather than on the magnitude or statistical significance of the pooled effects.

Subgroup analyses were conducted for peak VO_2_ and 6MWD, for which sufficient studies were available, according to the CR phase at intervention initiation, provision of real-time feedback, type of wearable device, type of exercise, and comparator characteristics. Subgroup analyses were performed using restricted maximum likelihood estimation with the Knapp-Hartung adjustment. Differences between subgroups were evaluated using formal tests for subgroup interaction. The comparator-based subgroup analysis was added during the peer-review process and was therefore considered post hoc. Given the limited number of studies within individual subgroups, the findings were interpreted cautiously. A diagnosis-based subgroup analysis (eg, post-PCI, post–coronary artery bypass grafting, or mixed populations of patients with cardiovascular disease) was not performed because several studies enrolled heterogeneous populations of patients with cardiovascular disease and too few studies were available within specific diagnostic categories to support reliable subgroup estimates.

Univariable random-effects meta-regression analyses were conducted for peak VO_2_ and 6MWD, for which sufficient study-level covariate data were available. These analyses were performed using restricted maximum likelihood estimation with the Knapp-Hartung adjustment. Mean participant age, proportion of female participants, and intervention duration were examined as potential moderators. Multivariable meta-regression was not performed because of the limited number of contributing studies. Meta-regression coefficients with 95% CIs and 2-sided *P* values were reported. Given the limited statistical power and use of aggregate study-level data, the meta-regression findings were interpreted as hypothesis-generating.

Sensitivity analyses were performed by excluding the cluster-randomized trial from the affected outcome-specific meta-analyses. A leave-one-out sensitivity analysis was additionally conducted for 6MWD by sequentially excluding each study and recalculating the pooled effect estimate and heterogeneity statistics. All analyses were performed using R version 4.5.2 (R Foundation for Statistical Computing) [28]. Statistical significance was defined as a 2-sided *P* value of <.05.

### Reporting Bias Assessment

Small-study effects were assessed for outcome-specific meta-analyses including at least 10 studies through visual inspection of funnel plots and the Egger regression test. Accordingly, these assessments were performed for peak VO_2_, which included 10 studies. Formal tests for funnel plot asymmetry were not performed for the remaining outcomes because fewer than 10 studies were available, resulting in limited statistical power and unreliable assessments of asymmetry. Funnel plot asymmetry was interpreted as potential evidence of small-study effects rather than definitive evidence of publication bias [26].

### Certainty Assessment

The certainty of evidence was assessed for all predefined primary and secondary outcomes using the Grading of Recommendations Assessment, Development and Evaluation (GRADE) approach [32]. The assessed outcomes included peak VO_2_, 6MWD, daily steps, AT, BMI, waist circumference, grip strength, and QoL. Four reviewers (BRK, JHC, S Young Lee, and KCS) participated in the assessment. Outcomes were independently evaluated in reviewer pairs using a structured Microsoft Excel form, and disagreements were resolved through discussion and consensus among all reviewers. Final judgments were summarized using GRADEpro guideline development tool [33].

The certainty of evidence for each outcome was evaluated across 5 domains: risk of bias, inconsistency, indirectness, imprecision, and publication bias. Because the evidence was derived from RCTs, the initial certainty was rated as high and was downgraded by 1 or 2 levels when serious or very serious concerns were identified within a domain. Downgrading decisions considered the outcome-level RoB 2 assessments, clinical and statistical heterogeneity, consistency in the direction and magnitude of effects, width of the 95% CIs, amount of available information, applicability to the review question, and evidence of possible publication bias or small-study effects. Prediction intervals were considered supplementary information when evaluating inconsistency but were not used as an independent criterion for downgrading. The overall certainty of evidence was categorized as high, moderate, low, or very low.

## Results

### Study Selection

The original database search identified 1428 records, and the updated search conducted on August 7, 2026, identified 552 records, resulting in a total of 1980 records identified through electronic databases. Three additional reports were identified through other sources across the original and updated searches. After removal of 633 duplicate database records, 1347 database records underwent title and abstract screening, of which 1249 were excluded.

The remaining 98 database reports were sought for retrieval and successfully retrieved for eligibility assessment. The 3 reports identified through other sources were also retrieved and assessed. Thus, a total of 101 reports were assessed for eligibility, and 81 were excluded for the following reasons: not RCTs (n=9), not meeting the eligibility criteria for study participants (n=7), not meeting the intervention criteria (n=14), protocol publications (n=30), overlapping or salami publications without distinct eligible data (n=7), or abstract-only reports without sufficient full-text data (n=14).

The remaining 20 publications, representing 18 unique trials, were included in the systematic review [20-23,34-49]. The updated database search resulted in the inclusion of Nishio et al [38], while the supplementary manual search resulted in the inclusion of Claes et al [39]. Of the 20 included reports, 19 reports representing 18 unique trials contributed outcomes eligible for the RoB 2 assessment [20-23,34-47,49], while 18 reports representing 17 unique studies contributed data to the quantitative meta-analysis [20-23,34-47].

Lahtio et al [35] and Hakala et al [45] were identified as companion publications arising from the same cluster-randomized trial (ISRCTN61225589). Both reports were retained because they provided distinct eligible outcomes: Lahtio et al [35] contributed data on 6MWD, BMI, and waist circumference, whereas Hakala et al [45] contributed data on daily steps. The reports were linked and treated as arising from a single underlying study.

Mitropoulos et al [23] and Ntovoli et al [48] were also identified as companion publications arising from the same trial cohort (NCT06071273). Ntovoli et al [48] did not report any of the outcomes predefined for this review and was therefore not included in the outcome-level RoB 2 assessment or quantitative meta-analysis. However, it was retained as a companion report in the systematic review and linked to Mitropoulos et al [23]. Accordingly, the cohort was counted as a single unique study.

One included study [49] did not contribute to the quantitative meta-analysis because it reported quality-adjusted life-years, a composite outcome incorporating both quality and duration of life that was not directly comparable with the conventional QoL outcomes reported by the other studies. Each unique study contributed only one effect estimate to any given outcome-specific meta-analysis; therefore, no participant was double-counted within the same pooled analysis. The study-selection process is presented in Figure 1.

**Figure 1:**
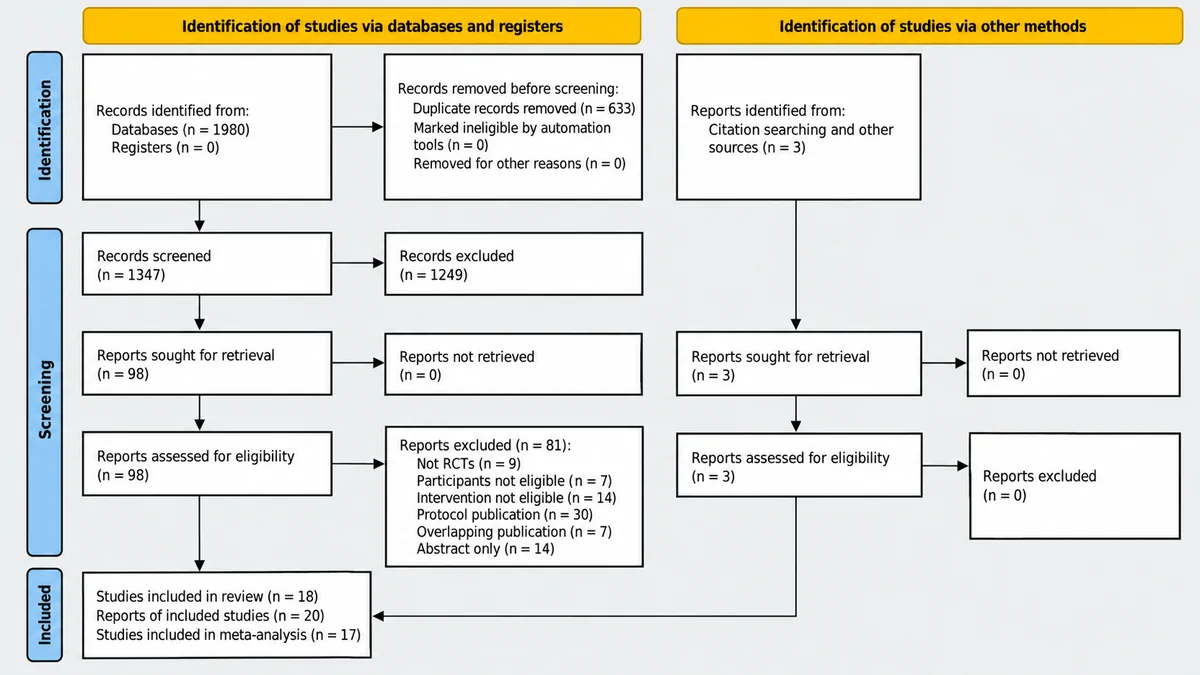
PRISMA (Preferred Reporting Items for Systematic Reviews and Meta-Analyses) 2020 flow diagram of study selection. The PRISMA diagram shows identification, screening, eligibility, and inclusion. The review contained 20 publications from 18 trials; 17 trials contributed to the meta-analysis. RCT: randomized controlled trial.

### Study Characteristics

Table 1 summarizes the characteristics of the 18 unique trials reported in 20 publications representing 18 unique trials, published between 2018 and 2025 [20-23,34-49]. At the unique-trial level, 6 studies were conducted in the United States [36,37,40,43,44,46], 2 each in China [20,21], the Czech Republic [34,41], and Japan [22,38], and 1 each in Greece [23,48], Finland [35,45], Uzbekistan [47], the Netherlands [49], and Belgium [42]. One multicenter study was conducted across Belgium and Ireland [39]. Companion publications from Greece [23,48] and Finland [35,45] were linked at the trial level, and each pair was counted only once in the country distribution.

**Table 1:** Characteristics of 20 publications representing 18 unique RCTs conducted across multiple countries and published between 2018 and 2025, evaluating wrist-worn wearable–guided CR delivered through fully remote or hybrid programs in adults with CVD, with interventions initiated during phase II or phase III CR.

Study, country	Study design	Population (intervention vs control)^a^	Intervention characteristics	Comparator type and components	Outcomes assessed^b^	Outcome assessment/follow-up
Zhang et al [20], (2025) China	Single-center RCT^c^	CHD^d^ (angina, MI^e^, PCI^f^); n=35 vs 35; dropout: 3 vs 5 (8/70, 11.4%); age: mean 62.67 (SD 4.89) years vs mean 57.2 (SD 13) years; male sex: 56.2% vs 63.3%	Timing: at discharge; delivery: home-based CR^g^ with wearable+app+ICT^h^; components: real-time feedback, remote supervision, PA^i^ and hemodynamic monitoring, medication reminders, educational content; device: smartwatch (Le-W20 Heart Health Watch); real-time feedback: yes; exercise: aerobic; dose: not reported; duration: 3 months	Usual care (standard HBCR^j^ without technological assistance)	Peak VO_2_^k^, AT^l^, QoL^m^	3 months
Zheng et al [21], (2024) China	Single-center RCT	CHD (PCI); n=55 vs 55; dropout: 2 vs 2 (4/110, 3.6%); age: mean 63.49 (SD 9.53) vs mean 63.04 (SD 10.44) years; male sex: 60% vs 57%	Timing: post discharge; delivery: home-based CR with wearable+app+ICT; components: remote supervision, PA and HR^n^ monitoring; device: smartband (model not reported); real-time feedback: no; exercise: aerobic; dose: 3-5 days/week, session duration not reported; duration: 3 months	Usual care (discharge instructions and a health guide handbook)	6MWD^o^, peak VO_2_, AT, QoL	3 months
Yagi et al [22], (2025) Japan	Single-center RCT	CVD^p^ (angina, MI, CHF^q^); n=23 vs 21; dropout: 4 vs 1 (5/44, 11.4%); age: 73 vs 76 years; male sex: 68% vs 95%	Timing: after outpatient center-based CR; delivery: home-based CR with wearable; components: PA and HR self-monitoring; device: smartwatch (Fitbit Versa 3); real-time feedback: no; exercise: aerobic, resistance; dose: 30 min/session, 5 days/week; duration: 3 months	Usual care (supervised hospital-based CR without wearable support)	Peak VO_2_, 6MWD, AT, grip strength	3 months
Mitropoulos et al [23], (2024) Greece	Single-center RCT	CAD^r^ (MI); n=18 vs 18; dropout: 3 vs 3 (6/36, 16.7%); age: mean 54.1 (SD 8.5) vs mean 53.5 (SD 6.6) years; male sex: 73% vs 73%	Timing: less than 4 weeks after MI; delivery: home-based CR with wearable+app+ICT; components: real-time feedback, remote supervision, PA and hemodynamic monitoring; device: smartwatch (ScanWatch); real-time feedback: yes; exercise: aerobic, resistance, balance, flexibility; dose: 60 min/session, 3 days/week; duration: 6 months	Community-based CR (supervised gym-based exercise without hemodynamic monitoring)	Peak VO_2_, daily steps	6 months
Wohlfahrt et al [34], (2024)^s^ Czech Republic	Single-center, cross-over RCT	MI; n=32 vs 32; dropout: 1 vs 2 (3/64, 4.7%); age: mean 51.6 (SD 10.2) vs mean 51.2 (SD 10.5) years; male sex: 90% vs 90%	Timing: 1 month after MI; delivery: home-based CR with wearable+app+ICT; components: remote supervision, PA monitoring; device: smartwatch (model not reported); real-time feedback: no; exercise: aerobic; dose: individualized daily step count target (derived from 6MWT^t^: 0.9 × 5 × 6MWT steps); duration: 3 months per period	Usual care (physical activity advice without wearable support)	Peak VO_2_, 6MWD, QoL	3 months for the meta-analysis (first period before crossover); total trial duration, 6 months
Lahtio et al [35], (2023)^u^ Finland	Single-center, cluster RCT	CHD (angina, MI, PCI, CABG^v^); n=29 (3 clusters) vs 30 (3 clusters); dropout: 3 vs 3 (6/59, 10.2%); age: mean 59.7 (SD 6.0) vs mean 59.2 (SD 6.1) years; male sex: 77% vs 86%	Timing: PCI or CABG 3-12 months prior to the study; delivery: home-based CR with wearable+app+ICT; components: remote supervision, PA and HR monitoring, educational content; device: smartband (Fitbit Charge); real-time feedback: no; exercise: aerobic, resistance, flexibility; dose: not reported; duration: 12 months	Usual care (conventional CR without remote technology)	6MWD, BMI, waist circumference, QoL	12 months
Beckie et al [36], (2023) United States	Multicenter RCT	CHD; n=23 vs 24; dropout: 1 vs 4 (5/47, 10.6%); age: mean 62.7 (SD 7.3) vs mean 59.7 (SD 10.5) years; male sex: 0% vs 0%	Timing: CHD with or without PCI within the prior 5 years; delivery: home-based CR with wearable+app+ICT; components: remote supervision, PA monitoring, educational content; device: smartwatch (Android model not reported); real-time feedback: no; exercise: aerobic, balance, flexibility; dose: not reported; duration: 3 months	Usual care (CR workbook/DVD with weekly telephone support)	6MWD, BMI, waist circumference, QoL	3 months
Lindman et al [37], (2021) United States	Multicenter RCT	TAVR^w^; n=25 vs 25; dropout: 0 (0%); age: mean 76 (SD 7) vs mean 76 (SD 9) years; male sex: 64% vs 68%	Timing: 1 month post-TAVR; delivery: home-based CR with wearable+app+ICT; components: remote supervision, PA and HR monitoring; device: smartband (Fitbit Alta HR); real-time feedback: no; exercise: aerobic, resistance; dose: not reported; duration: 6 weeks	Usual care (activity monitor without feedback or exercise instructions)	Daily steps, 6MWD, grip strength, QoL	6 weeks
Chokshi et al [40], (2018) United States	Multicenter RCT	CHD (angina, MI); n=50 vs 55; dropout: 1 vs 0 (1/105, 0.9%); age: mean 60 (SD 9.5) vs mean 59.1 (SD 11.5) years; male sex: 72.0% vs 67.3%	Timing: recent-onset ACS^x^ or coronary catheterization; delivery: home-based CR with wearable+app+ICT; components: remote supervision, PA monitoring; device: smartband (Misfit Shine); real-time feedback: no; exercise: aerobic; dose: not reported; duration: 16 weeks	Usual care (wearable step monitoring without personalized goals or feedback)	Daily steps	4 months
Batalik et al [41], (2020) Czech Republic	Single-center RCT	CVD (angina, MI, PCI, CABG); n=28 vs 28; dropout: 3 vs 2 (5/56, 8.9%); age: mean 56.5 (SD 6.9) vs mean 57.7 (SD 7.6) years; male sex: 80% vs 85%	Timing: CVD in the last 6 months; delivery: home-based CR with wearable+app+ICT; components: remote supervision, PA and HR monitoring; device: smartband (Polar M430); real-time feedback: no; exercise: aerobic; dose: 60 min/session, 3 days/week; duration: 3 months	Center-based CR (supervised outpatient exercise)	Peak VO_2_, QoL	3 months
Avila et al [42], (2020)^y^ Belgium	Single-center RCT	CAD; n=30 vs 30 vs 30; dropout: 4 vs 1 vs 5 (10/90, 11.1%); age: mean 62.2 (SD 7.1) vs mean 62.0 (SD 7.4) vs mean 63.7 (SD 7.4) years; male sex: 88% vs 90% vs 92%	Timing: after discharge from phase II center-based CR; delivery: home-based CR with wearable+app+ICT; components: remote supervision, PA and HR monitoring; device: smartwatch (Garmin Forerunner), sensewear mini armband (BodyMedia); real-time feedback: no; exercise: aerobic; dose: 30 min/session, 5 days/week; duration: 3 months	Center-based CR (supervised exercise); usual care (physical activity advice only)	Peak VO_2,_ daily steps, BMI, waist circumference, grip strength, QoL	12 months
Park et al [43], (2021) United States	Multicenter RCT	CVD; n=32 vs 28; dropout: 6 vs 3 (9/60, 15.0%); age: mean 66.7 (SD 8.6) vs mean 66.8 (SD 8.7) years; male sex: 73% vs 80%	Timing: after discharge from phase II center-based CR; delivery: home-based CR with wearable+app+ICT; components: remote supervision, PA and hemodynamic self-monitoring, educational content; device: smartband (Fitbit Charge); real-time feedback: no; exercise: aerobic; dose: not reported; duration: 2 months	Usual care (pedometer, step diary, and telephone support)	Daily steps, 6MWD	2 months
Duscha et al [44], (2018) United States	Single-center RCT	CVD (angina, MI, PCI, CABG, valve repair, CHF [4/25, 16%]); n=21 vs 11; dropout: 5 vs 2 (7/32, 21.9%); age: mean 59.9 (SD 8.1) vs mean 66.5 (SD 7.2) years; male sex: 81.2% vs 66.7%	Timing: after discharge from phase II center-based CR; delivery: home-based CR with wearable+app+ICT; components: remote supervision, PA monitoring; device: smartband (Fitbit Charge); real-time feedback: no; exercise: aerobic; dose: not reported; duration: 3 months	Usual care (physician-directed physical activity advice)	Peak VO_2_, daily steps	3 months
Hakala et al [45], (2021)^u^ Finland	Single-center, cluster RCT	CHD (angina, MI, PCI, CABG); n=29 vs 30 (same cohort as Lahtio et al[35]); dropout: 6/59 (10.2%); age: mean 59.7 (SD 6.0) vs mean 59.2 (SD 6.1) years; male sex: 76% vs 87%	Timing: CHD who attended the center-based CR program; delivery: home-based CR with wearable+app+ICT; components: remote supervision, PA and HR monitoring, educational content; device: wrist-worn smartband (Fitbit Charge), hip-worn accelerometer (Fitbit Zip); real-time feedback: no; exercise: aerobic; dose: not reported; duration: 12 months	Usual care (conventional CR without remote technology)	Daily steps	12 months
Dodson et al [46], (2025) United States	Multicenter RCT	CHD (MI, PCI, CABG); n=298 vs 102; dropout: 27 vs 17 (44/400, 11.0%); age: 71.0 vs 71.0 years; male sex: 72.5% vs 73.5%	Timing: hospitalized for AMI^z^, PCI, or CABG within prior 4 weeks; delivery: home-based CR with wearable+app+ICT; components: remote supervision, PA and HR monitoring; device: smartband (Fitbit Inspire); real-time feedback: no; exercise: aerobic; dose: 30 min/session, 5 days/week; duration: 3 months	Usual care (traditional ambulatory CR permitted)	6MWD, QoL	3 months
Madina et al [47], (2025) Uzbekistan	Single-center RCT	MI; n=150 vs 150; dropout: 0 (0%); age: mean 57.8 (SD 8.9) vs mean 59.1 (SD 9.5) years; male sex: 67.3% vs 62.6%	Timing: MI within the 3 months prior to study; delivery: home-based CR with wearable+app+ICT; components: remote supervision, PA and HR monitoring, early warning sign reporting, medication reminders, dietary advice; device: smartband (model not reported); real-time feedback: no; exercise: aerobic; dose: not reported; duration: 6 months	Standard CR (exercise program and monthly physiotherapist sessions without digital monitoring)	6MWD, QoL	6 months
Ntovoli et al [48], (2025)^aa^ Greece	Single-center RCT	CAD (MI); same trial as Mitropoulos et al [23]; age: 18-45 years (26.7%), 46-55 years (20.1%), 56-65 years (40%), >65 years (13.3%); male sex: 80%	Timing: less than 4 weeks after MI; delivery: home-based CR with wearable+app+ICT; components: real-time feedback, remote supervision, PA and hemodynamic monitoring; device: smartwatch (ScanWatch); real-time feedback: yes; exercise: aerobic, resistance, balance, flexibility; dose: 60 min/session, 3 days/week; duration: 6 months	Community-based CR (supervised gym-based exercise)	Perceived well-being (PERMA profiler), CR adherence, percentage of peak HR, RPE^ab^	6 months
Brouwers et al [49], (2021) Netherlands	Multicenter RCT	CAD (ACS, PCI); n=153 vs 147; dropout: 4 vs 12 lost to FU^ac^ (16/300, 5.3%); age: mean 60.8 (SD 9.3) vs mean 60.5 (SD 9.8) years; male sex: 87.6% vs 89.8%	Timing: at phase II of outpatient CR; delivery: home-based CR with wearable+app+ICT; components: remote supervision, PA and HR monitoring; device: smartwatch (Mio Alpha), hip-worn accelerometer (ActiGraph wGT3x-BT); real-time feedback: no; exercise: aerobic, functional, resistance; dose: not reported; duration: 3 months CTR^ad^+9 months relapse prevention	Center-based CR (supervised group-based exercise)	QoL	12 months
Nishio et al [38], (2025) Japan	Single-center RCT	CAD (PCI, CABG); n=25 vs 25; dropout: 1 vs 0 lost to FU (1/50, 2.0%); age: mean 63.8 (SD 6.4) vs mean 62.6 (SD 7.9) years; male sex: 95.8% vs 92%	Timing: outpatient phase III CR candidates, after baseline CPET^ae^; delivery: home-based CR with wearable+app+ICT; components: real-time PA and HR monitoring, remote supervision, weekly individualized text-message coaching, monthly videoconferencing; device: smartwatch (Fitbit Sense); real-time feedback: yes; exercise: aerobic; dose: ≥30 min/session, 5 days/week; duration: 3 months	Wearable-supported CPET-based home exercise without online coaching	Peak VO_2_, AT, daily steps, BMI	3 months
Claes et al [39], (2020) Belgium	Multicenter RCT	CVD (PCI/CABG/valve repair); n=60 vs 60; dropout: 7 vs 13 lost to FU (20/120, 16.7%); age: mean 61.7 (SD 14.5) vs mean 59.6 (SD 13.2) years; male sex: 81.7% vs 81.7%	Timing: initiated during the final 4 weeks of center-based phase II CR and continued into phase III; delivery: home-based CR with wearable+app+ICT; components: remote supervision, PA and HR monitoring, telecoaching, social networking, automated supportive text messages or emails; device: smartband (Microsoft Band 2); real-time feedback: no; exercise: aerobic; dose: 150 min/week moderate-intensity PA; duration: 6 months	Usual care (physical activity advice without structured CR)	Peak VO_2_, daily steps, grip strength, BMI, QoL	6 months

^a^Unless otherwise indicated, values are presented as intervention vs control. For studies with more than 2 arms, values are presented in the order of the study arms.

^b^Measurement methods and units: peak VO_2_ and VO_2_ max were measured using cardiopulmonary exercise testing (CPET) and expressed in mL/kg/min. AT was determined during CPET and expressed in mL/kg/min. The 6-minute walk distance was measured using the 6-minute walk test and expressed in meters. Physical activity was assessed using the wearable device or accelerometer specified in each study and reported as steps/day, minutes/day, or minutes/week. Heart rate was expressed as beats/min; blood pressure, as mmHg; body weight, as kg; BMI, as kg/m2; waist, hip, and calf circumferences, as cm; grip strength, as kg; left ventricular ejection fraction, as a percentage; and glycated hemoglobin, as a percentage. Quality of life and other patient-reported outcomes were assessed using the validated instruments specified in the original studies.

^c^RCT: randomized controlled trial.

^d^CHD: coronary heart disease.

^e^MI: myocardial infarction.

^f^PCI: percutaneous coronary intervention.

^g^CR: cardiac rehabilitation.

^h^ICT: information and communication technology.

^i^PA: physical activity.

^j^HBCR: home-based cardiac rehabilitation.

^k^VO_2_: oxygen consumption.

^l^AT: anaerobic threshold.

^m^QoL: quality of life.

^n^HR: heart rate.

^o^6MWD: 6-minute walk distance.

^p^CVD: cardiovascular disease.

^q^CHF: congestive heart failure.

^r^CAD: coronary artery disease.

^s^Wohlfahrt et al [34] used a crossover design. Data from the first 3-month period before crossover were used for the meta-analysis to avoid potential carryover effects. The 64 participants were randomized to 2 treatment sequences (32 vs 32).

^t^6MWT: 6-minute walk test.

^u^Lahtio et al [35] and Hakala et al [45] are companion publications arising from the same cluster-randomized trial (ISRCTN61225589). They reported different eligible outcomes and were treated as one unique underlying trial for study-level counting, including the country distribution. No participant was counted more than once within any outcome-specific meta-analysis.

^v^CABG: coronary artery bypass grafting.

^w^TAVR: transcatheter aortic valve replacement.

^x^ACS: acute coronary syndrome.

^y^Avila et al [42] used a 3-arm design. For each outcome-specific meta-analysis, only the eligible wearable-supported intervention arm and the corresponding usual-care comparator were included; no comparator group was entered more than once.

^z^AMI: acute myocardial infarction.

^aa^Ntovoli et al [48] and Mitropoulos et al [23] are companion publications arising from the same randomized trial (NCT06071273). Ntovoli et al did not report any of the outcomes predefined for this review and therefore did not contribute to the outcome-level Risk of Bias 2 assessment or quantitative meta-analysis. The reports were linked and treated as one unique underlying trial for study-level counting, including the country distribution.

^ab^RPE: rating of perceived exertion.

^ac^FU: follow-up.

^ad^CTR: cardiac telerehabilitation.

^ae^CPET: cardiopulmonary exercise testing.

Lahtio et al [35] and Hakala et al [45] were companion publications arising from the same cluster-randomized trial (ISRCTN61225589), while Mitropoulos et al [23] and Ntovoli et al [48] were companion publications arising from the same randomized trial (NCT06071273). Each companion-publication pair was linked and treated as representing one unique underlying trial for study-level counting, including the country distribution.

Of the 18 unique trials, 10 were single-center RCTs [20-23,34,38,41,42,44,47], 7 were multicenter RCTs [36,37,39,40,43,46,49], and 1 was a cluster RCT reported in 2 companion publications [35,45]. Wohlfahrt et al [34] used a crossover RCT design, whereas the remaining individually randomized studies used parallel-group designs.

All eligible intervention groups implemented remote or hybrid CR incorporating a wrist-worn wearable device as a core intervention component [20-23,34-49]. The wearable technologies included smartbands [21,35,37,39-47] and smartwatches [20,22,23,34,36,38,48,49]. A small number of studies incorporated additional sensors for intervention delivery or outcome assessment [39,45,49]. Nishio et al [38] used a Fitbit Sense integrated with a real-time monitoring platform and compared additional online coaching with wearable-supported CR without coaching. Claes et al [39] evaluated the PATHway technology-enabled home-based CR platform, which incorporated a Microsoft Band 2, remotely delivered exercise sessions, physical activity monitoring, automated feedback, education, and behavioral support.

The comparator interventions included usual care, home-based exercise without wearable-guided support, passive or minimally supported wearable monitoring, and structured center- or community-based CR [20-23,34-49]. Thus, the presence of a wearable device was not necessarily exclusive to the intervention group. Rather, the eligible intervention groups actively integrated wearable-derived data into CR delivery through monitoring, feedback, coaching, exercise guidance, or adherence management.

In terms of intervention components, nearly all studies included remote supervision and physical activity monitoring [20,21,23,34-49]. Many studies also incorporated HR or hemodynamic monitoring [20-23,35,37-39,41-43,45-49], whereas real-time or individualized feedback was provided in a subset of studies [20,23,38,39,48]. Additional components included educational or behavioral-support content [20,35,36,39,43,45,47] and warning systems for abnormal physiological signals [20,47].

The exercise programs were predominantly aerobic, including the intervention evaluated by Nishio et al [38], although several studies implemented combined training incorporating aerobic and resistance exercise [22,23,35,37,39,48,49]. Some interventions additionally included balance or flexibility exercises [23,35,36,39,48]. When reported, the prescribed exercise dose generally ranged from 30 to 60 minutes per session, performed 3-5 times per week [22,23,34,38,41,42,46,48].

Intervention duration ranged from 6 weeks to 12 months, with most interventions lasting approximately 3-6 months [20-23,34,36,38-42,44,46-49]. Follow-up assessments were generally aligned with intervention completion, although some studies reported extended follow-up to 12 months [42,46,49]. When multiple assessment time points were available, the assessment conducted at or closest to intervention completion was preferentially selected for quantitative synthesis. When no assessment was reported at intervention completion, the earliest eligible postintervention assessment was used. In Avila et al [42], the relevant outcomes were available at the 12-month follow-up following a 3-month intervention.

Overall, the 18 unique trials randomized 1993 participants with CVD receiving wrist-worn wearable–guided CR through fully remote or hybrid programs initiated during phase II or phase III CR. Of the 20 included publications, 18 publications representing 17 unique trials contributed data to at least 1 quantitative meta-analysis [20-23,34-47]. Because the number of participants contributing data differed across outcomes and assessment time points, outcome-specific sample sizes are reported with each meta-analysis. Across the included studies, mean participant age ranged from approximately 51 to 76 years, and the proportion of male participants varied substantially, ranging from 0% to approximately 96%; 1 study enrolled only female participants [36]. Individual study sample sizes ranged from 25 to 400 participants. Most participants had coronary artery disease or had undergone PCI or cardiac surgery.

### Risk of Bias in Studies

Among the 20 publications representing 18 unique trials, 19 reports were included in the outcome-level RoB assessment. Ntovoli et al [48], a companion publication from the same trial as Mitropoulos et al [23], was not assessed because it did not report any of the predefined outcomes of interest. RoB was assessed separately for each outcome using the standard or design-specific Cochrane RoB 2 tool, as appropriate.

For peak VO_2_, 4 of the 10 contributing studies were rated as having low overall RoB and 6 as having some concerns [20-23,34,38,39,41,42,44], with no studies rated as having high RoB. For 6MWD, 8 of the 9 contributing studies were rated as having some concerns and 1 as having high RoB [21,22,34-37,43,46,47]. All studies contributing to BMI [35,36,38,39,42] and waist circumference [35,36,42] were rated as having some concerns. For AT, 2 of the 4 contributing studies were rated as having low RoB and 2 as having some concerns [20-22,38]. For grip strength, 3 of the 4 contributing studies were rated as having some concerns and 1 as having high RoB [22,37,39,42].

In contrast, substantial RoB concerns were observed for daily steps and QoL. For daily steps, 7 of the 9 studies were rated as having high overall RoB [37,38,40,42-45], primarily because of bias due to deviations from intended interventions. For QoL, 10 of the 13 studies were rated as having high overall RoB [20,34-36,38,39,42,46,47,49], primarily because of bias in outcome measurement. The outcome-specific RoB assessments are summarized in Multimedia Appendix 3.

### Results of Individual Studies

Outcome-specific summary statistics and effect estimates for each included study are presented in the forest plots (Figures 2-9) and the corresponding supplementary tables (Multimedia Appendix 4). For continuous outcomes, individual study effects are reported as MDs with 95% CIs for peak VO_2_, 6MWD, daily steps, AT, BMI, waist circumference, and grip strength. For QoL, individual study effects are reported as SMDs using Hedges *g* with 95% CIs because different validated instruments were used across studies. The number of participants contributing data to each outcome-specific effect estimate is also presented. Individual study findings varied in magnitude and, for several outcomes, in direction, as reflected in the forest plots and the between-study heterogeneity reported in the Results of Syntheses section.

**Figure 2:**
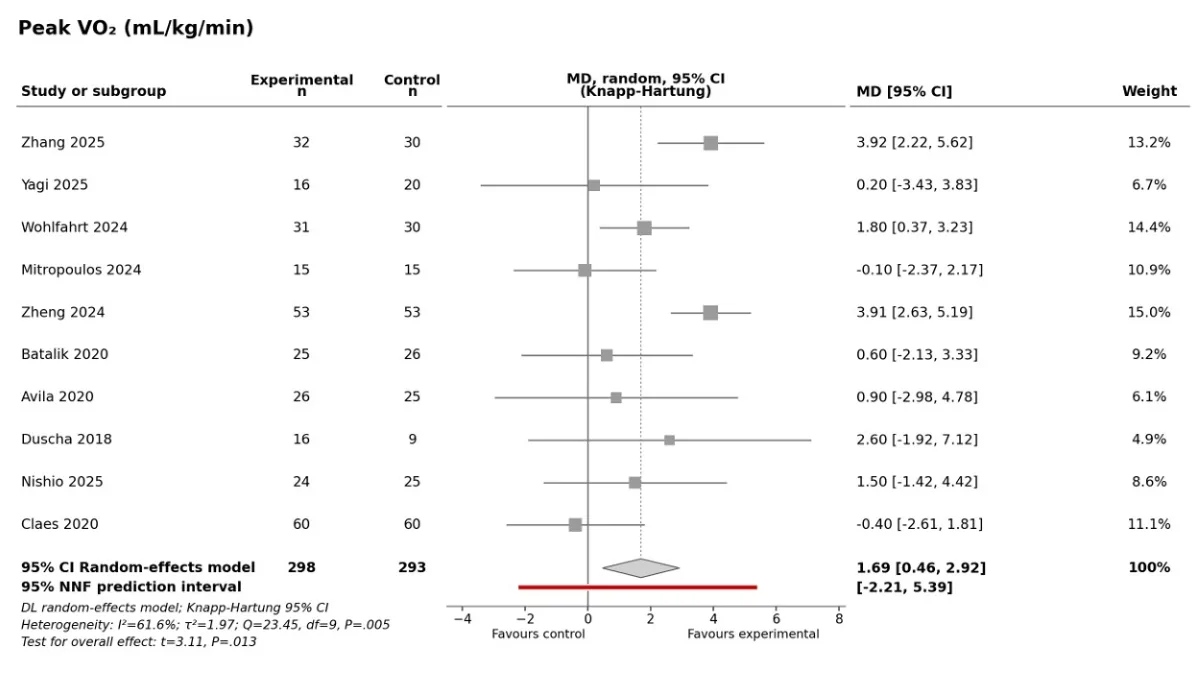
Random-effects meta-analysis of peak oxygen consumption (VO2). Study and pooled random-effects mean differences (MDs) are shown with 95% CIs. DerSimonian-Laird (DL) estimated between-study variance. Pooled CIs used the Knapp-Hartung adjustment. The labeled normal-normal framework (NNF) 95% prediction interval represents a future comparable study.

### Results of Syntheses

#### Overview

For each outcome, the pooled estimate and 95% CI represent the estimated average effect across the included studies, whereas the 95% prediction interval describes the expected distribution of true effects across comparable clinical settings while accounting for between-study heterogeneity.

#### Primary Outcomes

##### Peak VO2

A total of 10 studies involving 591 participants were included in the meta-analysis of peak VO_2_ [20-23,34,38,39,41,42,44]. On average, wrist-worn wearable–guided CR delivered through fully remote or hybrid programs was associated with greater peak VO_2_ than the control interventions (MD 1.69, 95% CI 0.46-2.92 mL/kg/min; *P*=.01). Between-study variability was reflected by τ^2^=1.97; *I*^2^=61.6%; and Cochran Q=23.45 (*P*=.005). The NNF 95% prediction interval ranged from –2.21 to 5.39 mL/kg/min and included the null. Thus, although the estimated average effect favored the intervention, a benefit may not be observed consistently across future comparable settings (Figure 2).

##### 6MWD

A total of 9 studies involving 1094 participants were included in the meta-analysis of 6MWD [21,22,34-37,43,46,47]. The pooled estimate indicated greater average 6MWD in the intervention groups than in the control groups (MD 31.03, 95% CI 7.66-53.92 m; *P*=.02). Between-study variability was reflected by τ^2^=1241.17; *I*^2^=87.8%; and Cochran Q=65.78 (*P*<.001). The NNF 95% prediction interval was wide and included the null (–62.11 to 123.18 m), indicating uncertainty in the effect expected in a future comparable setting despite the favorable average effect (Figure 3).

**Figure 3:**
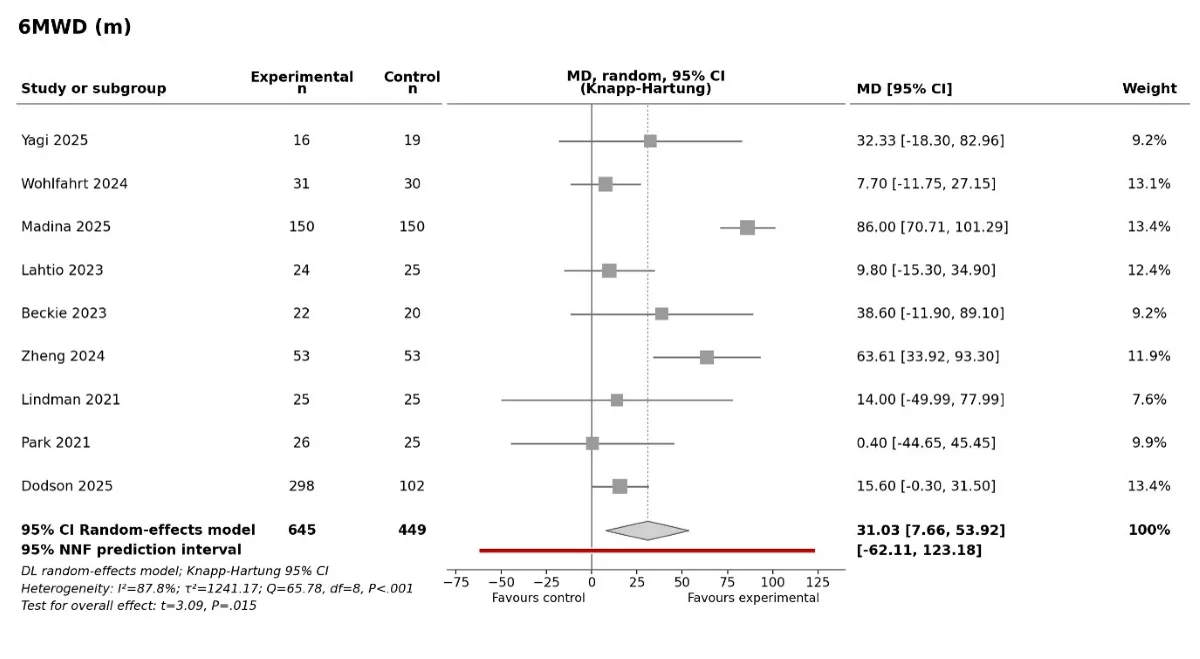
Random-effects meta-analysis of 6-minute walk distance (6MWD). Study and pooled random-effects mean differences (MDs) for 6MWD are shown with 95% CIs. DerSimonian-Laird (DL) estimated variance, pooled CIs used Knapp-Hartung adjustment, and the labeled normal-normal framework (NNF) 95% prediction interval represents a future comparable study.

##### Daily Steps

A total of 9 studies reported daily steps. Avila et al [42] was excluded from quantitative synthesis because the report did not provide sufficient numerical information to calculate a daily-step effect estimate and its variance. The remaining 8 studies involving 474 participants were included [23,37-40,43-45]. On average, the intervention groups accumulated more daily steps than the control groups (MD 1062.8, 95% CI 383.5-1825.0 steps/day; *P*=.01). Between-study variability was reflected by τ^2^=193,015.86; *I*^2^=30.0%; and Cochran Q=10.00 (*P*=.19). The NNF 95% prediction interval included the null (–503.0 to 2860.0 steps/day), suggesting that a benefit may not be observed consistently across future comparable settings (Figure 4).

**Figure 4:**
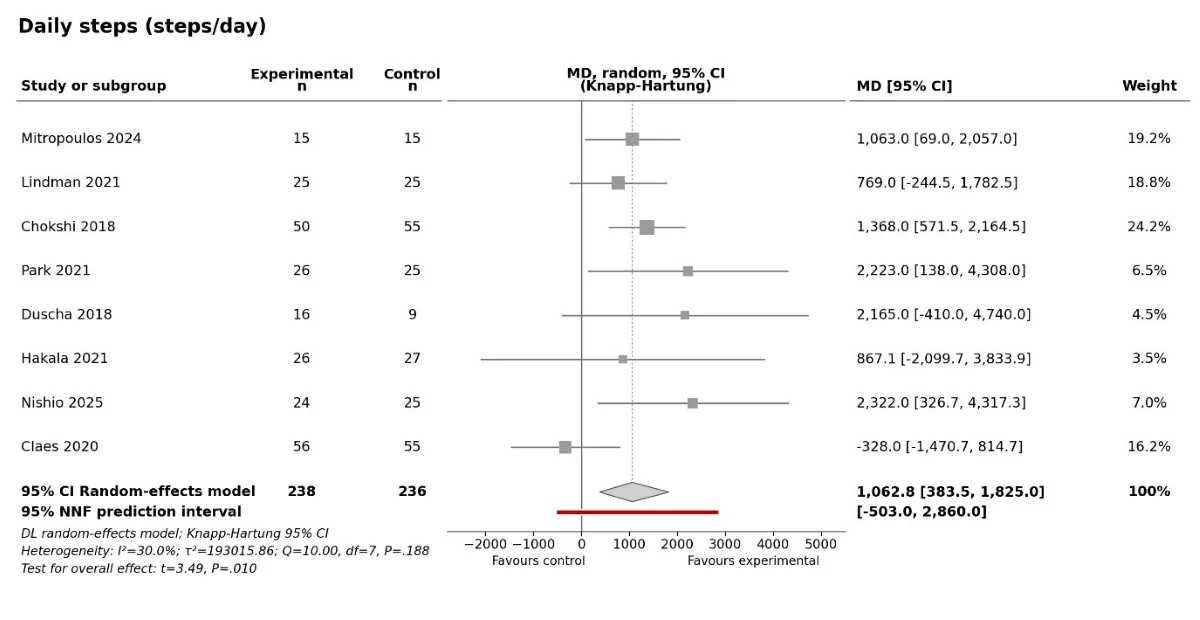
Random-effects meta-analysis of daily steps. Study and pooled random-effects mean differences (MDs) in daily steps are shown with 95% CIs. DerSimonian-Laird (DL) estimated variance, pooled CIs used the Knapp-Hartung adjustment, and the labeled normal-normal framework (NNF) 95% prediction interval represents a future comparable study.

#### Secondary Outcomes

##### AT

A total of 4 studies involving 256 participants were included in the meta-analysis of AT [20-22,38]. The pooled estimate favored the intervention but was imprecise and included the null after Knapp-Hartung adjustment (MD 1.02, 95% CI –0.22 to 2.21 mL/kg/min; *P*=.08). Between-study variability was reflected by τ^2^=0.27; *I*^2^=35.0%; and Cochran Q=4.62 (*P*=.20). The NNF 95% prediction interval also included the null (–2.05 to 3.65 mL/kg/min), providing no clear evidence of a consistent benefit (Figure 5).

**Figure 5:**
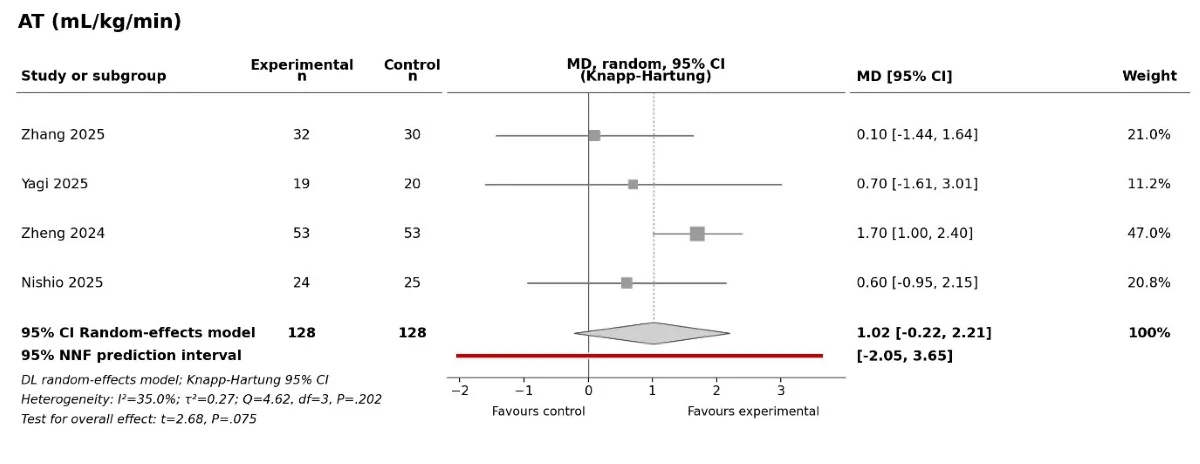
Random-effects meta-analysis of anaerobic threshold (AT). Study and pooled random-effects mean differences (MDs) in AT are shown with 95% CIs. DerSimonian-Laird (DL) estimated variance, pooled CIs used the Knapp-Hartung adjustment, and the labeled normal-normal framework (NNF) 95% prediction interval represents a future comparable study.

##### BMI

A total of 5 studies involving 314 participants were included in the meta-analysis of BMI [35,36,38,39,42]. The pooled estimate suggested a possible reduction but was imprecise and included the null after Knapp-Hartung adjustment (MD –0.67, 95% CI –1.64 to 0.24 kg/m^2^; *P*=.12). Between-study variability was reflected by τ^2^=0.11; *I*^2^=17.1%; and Cochran Q=4.82 (*P*=.31). The NNF 95% prediction interval included the null (–3.03 to 1.60 kg/m^2^), providing no clear evidence of a consistent reduction (Figure 6).

**Figure 6:**
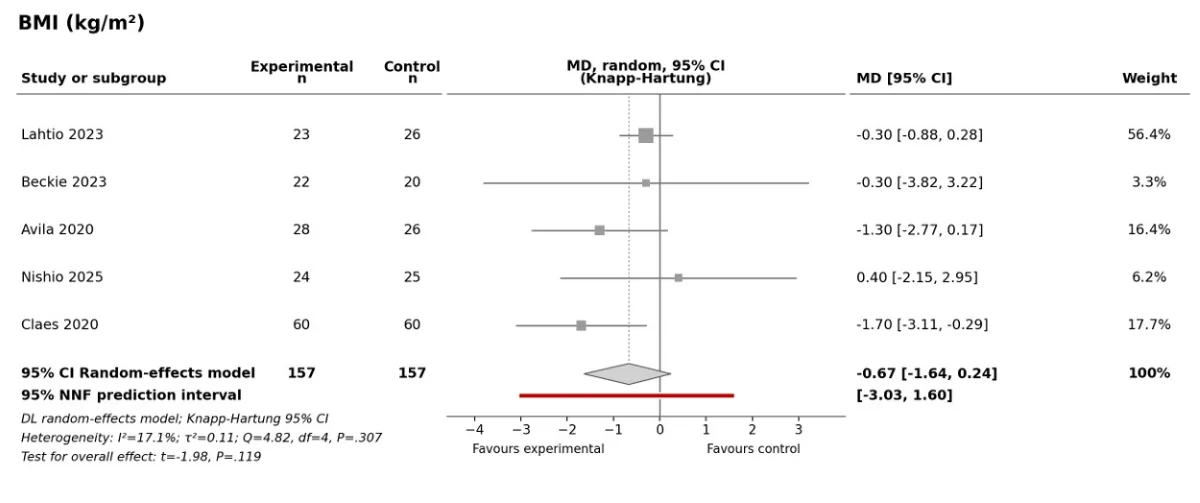
Random-effects meta-analysis of BMI. Study and pooled random-effects mean differences (MDs) in BMI are shown with 95% CIs. DerSimonian-Laird (DL) estimated variance, pooled CIs used Knapp-Hartung adjustment, and the labeled normal-normal framework (NNF) 95% prediction interval represents a future comparable study.

##### Waist Circumference

A total of 3 studies involving 145 participants were included in the meta-analysis of waist circumference [35,36,42]. The pooled estimate suggested a possible reduction but was imprecise and included the null after Knapp-Hartung adjustment (MD –2.48, 95% CI –5.90 to 0.17 cm; *P*=.07). The estimated between-study variance was zero (τ^2^=0; *I*^2^=0%; Cochran Q=0.77; *P*=.68), but this estimate was based on only 3 studies and should not be interpreted as evidence of identical effects. The NNF 95% prediction interval included the null (–9.76 to 3.35 cm), providing no clear evidence of a consistent reduction (Figure 7).

**Figure 7:**
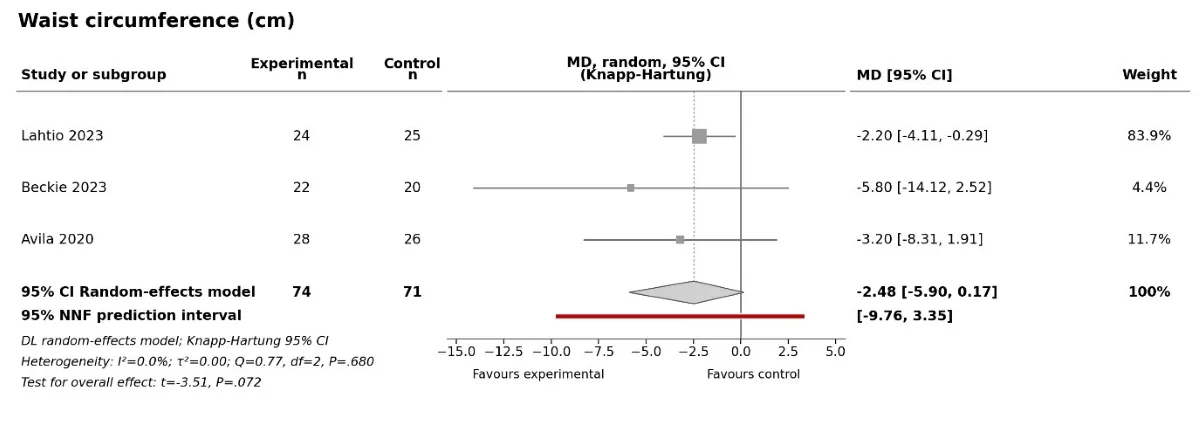
Random-effects meta-analysis of waist circumference. Study and pooled random-effects mean differences (MDs) in waist circumference are shown with 95% CIs. DerSimonian-Laird (DL) estimated variance, pooled CIs used the Knapp-Hartung adjustment, and the labeled normal-normal framework (NNF) 95% prediction interval represents a future comparable study.

##### Grip Strength

A total of 4 studies involving 236 participants were included in the meta-analysis of grip strength [22,37,39,42]. The pooled analysis showed no evidence of an average between-group difference (MD –0.06, 95% CI –1.24 to 1.08 kg; *P*=.87). The estimated between-study variance was zero (τ^2^=0; *I*^2^=0%; Cochran Q=0.29; *P*=.96), although only 4 studies contributed. The NNF 95% prediction interval also included the null (–1.45 to 1.28 kg), providing no evidence of a consistent benefit (Figure 8).

**Figure 8:**
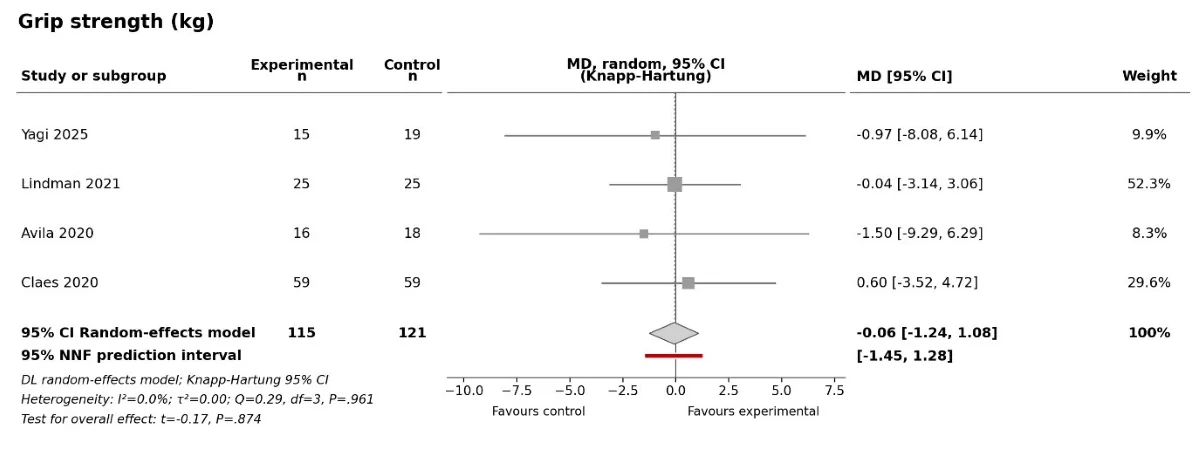
Random-effects meta-analysis of grip strength. Study and pooled random-effects mean differences (MDs) in grip strength are shown with 95% CIs. DerSimonian-Laird (DL) estimated variance. Pooled CIs used Knapp-Hartung adjustment. The labeled normal-normal framework (NNF) 95% prediction interval represents a future comparable study.

##### QoL

Although 13 studies reported QoL outcomes, only 5 studies involving 580 participants reported a valid overall or composite QoL score with sufficient numerical data for quantitative synthesis [34,37,39,41,47]. Zhang et al [20] was excluded because the reported SF-36 total score was not a validated summary score and was inconsistent with the stated 0-100 scoring range. The exploratory synthesis showed no clear average between-group difference (SMD 0.42, 95% CI –0.49 to 1.34; *P*=.27). Marked between-study variability remained (τ^2^=0.85; *I*^2^=95.0%; Cochran Q=80.09; *P*<.001), and a reliable NNF 95% prediction interval was not reported. Given the clinical and statistical variability across instruments and outcome domains, the pooled estimate was interpreted cautiously, and findings were considered primarily descriptive (Figure 9).

**Figure 9:**
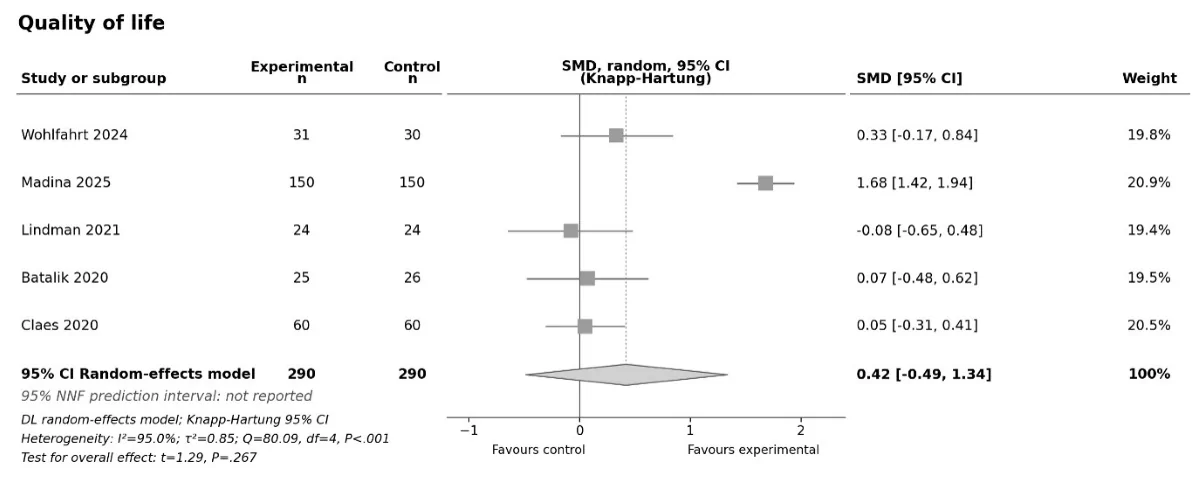
Random-effects meta-analysis of QoL. Study and pooled random-effects Hedges g standardized mean differences (SMDs) are shown with 95% CIs, and pooled CIs used Knapp-Hartung adjustment. Zhang et al [20] was excluded because its 36-Item Short Form Health Survey total was not validated. No reliable normal-normal framework (NNF) prediction interval was reported because variability remained marked. DL: DerSimonian-Laird.

### Subgroup Analyses

#### Peak VO2

Subgroup analyses for peak VO_2_ were conducted according to the CR phase at intervention initiation, provision of real-time feedback, wearable-device type, exercise type, and comparator characteristics. With restricted maximum likelihood estimation and Knapp-Hartung-adjusted subgroup CIs, the average effect was numerically larger in studies in which CR was initiated during phase II (MD 2.51, 95% CI –0.46 to 5.47 mL/kg/min; 4 studies) than in those initiated during phase III (MD 0.58, 95% CI –0.44 to 1.61 mL/kg/min; 6 studies). However, the formal Knapp-Hartung interaction test did not demonstrate a statistically significant difference between the subgroups (interaction: *P*=.08).

The average effect was also numerically larger in studies using aerobic exercise alone (MD 2.06, 95% CI 0.67-3.44 mL/kg/min; 8 studies) than in studies using combined aerobic and resistance exercise (MD –0.02, 95% CI –1.73 to 1.70 mL/kg/min; 2 studies); however, the Knapp-Hartung subgroup difference was not statistically significant (interaction: *P*=.16).

No statistically significant subgroup differences were identified according to provision of real-time feedback (interaction: *P*=.75), wearable-device type (interaction: *P*=.99), or comparator characteristics (interaction: *P*=.73). Overall, none of the formal interaction tests reached statistical significance (Figure 10). The numerically larger effects observed with phase II initiation and aerobic-only exercise should therefore be considered hypothesis-generating, particularly given the small number of studies within some subgroups.

**Figure 10:**
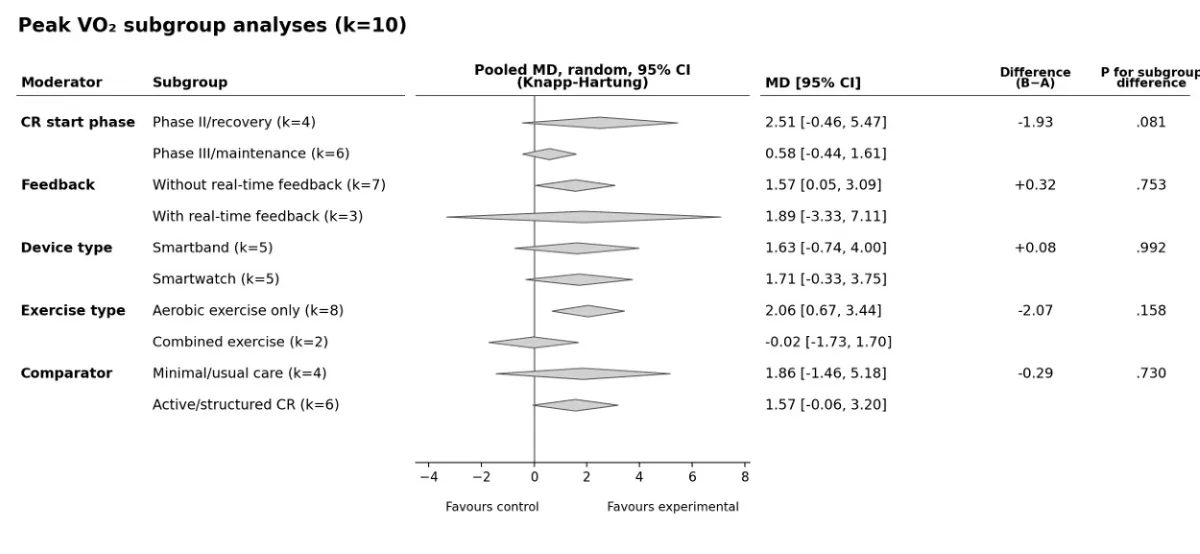
Subgroup analyses of peak oxygen consumption (VO2). Random-effects subgroup estimates used restricted maximum likelihood and Knapp-Hartung 95% CIs. No interaction was statistically significant (cardiac rehabilitation [CR] phase: P=.08; exercise type: P=.16). MD: mean difference.

#### 6MWD

Subgroup analyses for 6MWD were conducted according to the CR phase at intervention initiation, wearable-device type, exercise type, and comparator characteristics. With restricted maximum likelihood estimation and Knapp-Hartung-adjusted subgroup CIs, the pooled MD was 39.26 (95% CI –5.54 to 84.07 m; 5 studies) for interventions initiated during phase II and 15.20 (95% CI –8.68 to 39.08 m; 4 studies) for interventions initiated during phase III (interaction: *P*=.35). The pooled MD was 34.50 (95% CI –3.00 to 72.01 m; 6 studies) for smartbands and 16.23 (95% CI –23.10 to 55.56 m; 3 studies) for smartwatches (interaction: *P*=.63). For aerobic exercise alone and combined exercise, the pooled MDs were 36.05 (95% CI –11.12 to 83.22 m; 5 studies) and 17.88 (95% CI –3.60 to 39.35 m; 4 studies), respectively (interaction: *P*=.55). For minimal or usual-care and active or structured CR comparators, the pooled MDs were 24.08 (95% CI –0.80 to 48.97 m; 6 studies) and 43.20 (95% CI –60.40 to 146.81 m; 3 studies), respectively (interaction: *P*=.38). None of the interaction tests were statistically significant (Figure 11).

**Figure 11:**
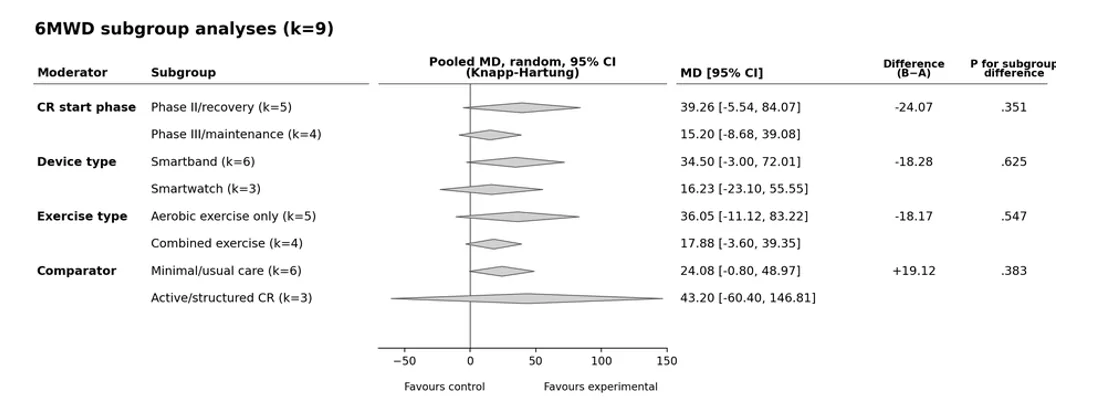
Subgroup analyses of 6-minute walk distance (6MWD). Random-effects subgroup estimates for 6MWD used restricted maximum likelihood and Knapp-Hartung 95% CIs. No interaction was statistically significant. CR: cardiac rehabilitation; MD: mean difference.

Given the limited number of studies within several subgroups, all subgroup findings were interpreted cautiously. The comparator-based subgroup analyses were conducted post hoc in response to peer review. Prediction intervals were not calculated for subgroup analyses because subgroup-specific estimates were based on a limited number of studies. Study-level results for the peak VO_2_ and 6MWD subgroup analyses are provided in Figures S1-S9 in Multimedia Appendix 4.

### Meta-Regression Analyses

#### Peak VO2

Univariable random-effects meta-regression analyses were conducted across the 10 studies contributing to the peak VO_2_ meta-analysis to examine associations with the proportion of female participants, intervention duration, and mean participant age.

A higher proportion of female participants was associated with a larger intervention effect on peak VO_2_ (β=.088, 95% CI 0.016-0.160 mL/kg/min per 1-percentage-point increase in the proportion of female participants; *P*=.02). Thus, at the study level, a 10-percentage-point increase in the proportion of female participants corresponded to an estimated 0.88-mL/kg/min larger MD in peak VO_2_. This finding should be interpreted cautiously because the analysis was based on aggregate study-level data and does not establish an individual-level sex-specific treatment effect.

Intervention duration showed a negative association with the intervention effect, but the association did not reach the prespecified 2-sided significance threshold (β=–.156, 95% CI –0.346 to 0.034 mL/kg/min per additional week; *P*=.10). Mean participant age was not associated with the intervention effect (β=.021, 95% CI –0.211 to 0.252 mL/kg/min per additional year; *P*=.84). The peak VO_2_ meta-regression results are presented in Figure 12.

**Figure 12:**
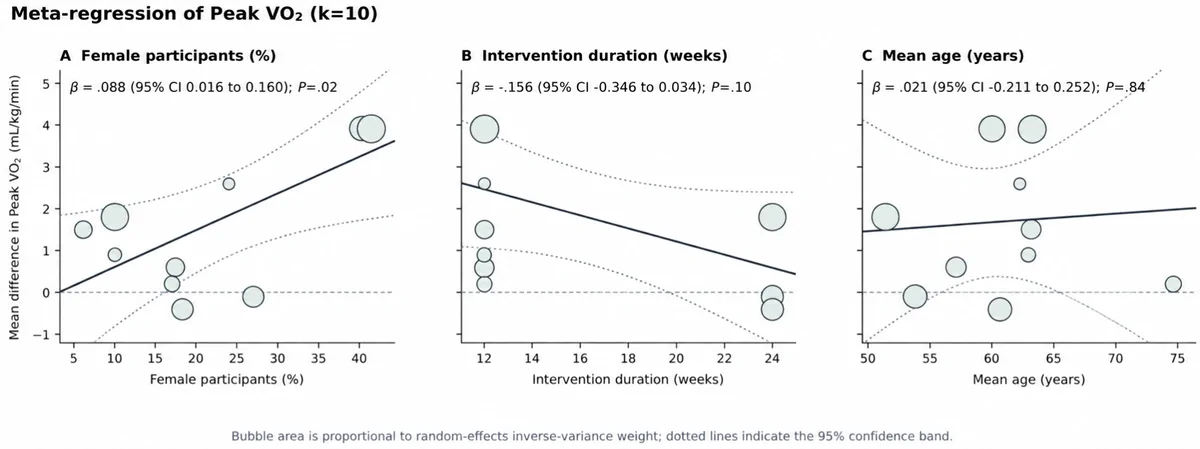
Univariable meta-regression analyses of peak oxygen consumption (VO2). Bubble plots show random-effects meta-regression of peak VO2 mean difference against female proportion, intervention duration, and mean age across 10 studies; area reflects inverse-variance weight; and dotted lines show 95% confidence bands.

#### 6MWD

Univariable random-effects meta-regression analyses were conducted across the 9 studies contributing to the 6MWD meta-analysis. No statistically significant associations were identified for any of the examined study-level moderators. The proportion of female participants was not significantly associated with the intervention effect on 6MWD (β=.453, 95% CI –0.576 to 1.482 m per 1-percentage-point increase; *P*=.33). Intervention duration was also not associated with the effect estimate (β=–.139, 95% CI –2.156 to 1.878 m per additional week; *P*=.88), nor was mean participant age (β=–.676, 95% CI –4.005 to 2.653 m per additional year; *P*=.65). The 6MWD meta-regression results are presented in Figure 13.

**Figure 13:**
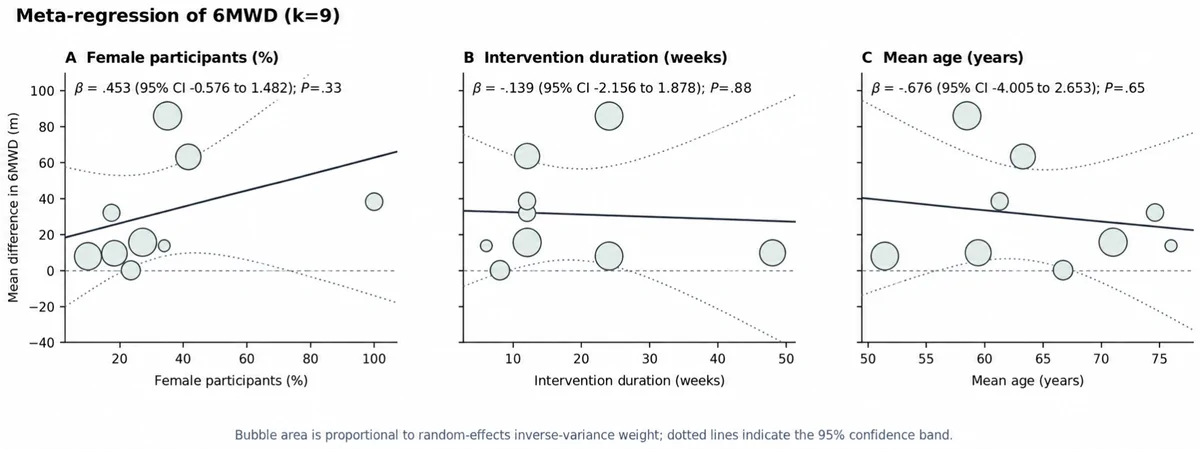
Univariable meta-regression analyses of 6-minute walk distance (6MWD). Bubble plots show random-effects meta-regression of 6MWD mean difference against female proportion, intervention duration, and mean age across 9 studies; area reflects inverse-variance weight; and dotted lines show 95% confidence bands.

Given the small number of contributing studies, limited statistical power, and use of aggregate study-level covariates, all meta-regression findings were considered exploratory and hypothesis-generating. In particular, the association between the proportion of female participants and the peak VO_2_ effect should not be interpreted as evidence that individual female participants experienced greater benefits.

### Sensitivity Analyses

#### Exclusion of the Cluster-Randomized Trial

Sensitivity analyses excluding the cluster-randomized trial were conducted for 6MWD, daily steps, BMI, and waist circumference, which were the outcomes to which the trial contributed.

For 6MWD, exclusion of the cluster-randomized trial did not materially change the pooled average effect (MD 33.95, 95% CI 7.60-60.30 m; *P*=.02). Between-study variability remained (*I*^2^=88.4%; τ^2^=1343.55), and the NNF 95% prediction interval included the null (–67.52 to 135.99 m).

For daily steps, the pooled average effect remained statistically significant after exclusion of the cluster-randomized trial (MD 1084.5, 95% CI 294.6-1961.1 steps/day; *P*=.02). Between-study variability was reflected by *I*^2^=39.9% and τ^2^=265,305.58, and the NNF 95% prediction interval included the null (–704.8 to 3077.3 steps/day).

For BMI, exclusion of the cluster-randomized trial resulted in a larger estimated reduction, but the Knapp-Hartung-adjusted CI included the null (MD –1.18, 95% CI –2.48 to 0.35 kg/m^2^; *P*=.08; *I*^2^=0%; τ^2^=0). The NNF 95% prediction interval also included the null (–3.68 to 1.71 kg/m^2^).

For waist circumference, exclusion of the cluster-randomized trial moved the point estimate farther from the null (from –2.48 to –3.91 cm), but precision decreased because only 2 studies remained (MD –3.91, 95% CI –8.27 to 0.44 cm; *P*=.08; *I*^2^=0%). An NNF prediction interval was not reported because only 2 studies remained, below the number for which the method has demonstrated adequate performance. Thus, the estimated effect became larger in magnitude, but the evidence became less precise and remained sensitive to a single-study exclusion.

#### Leave-One-Out Analysis for 6MWD

A leave-one-out sensitivity analysis was conducted for 6MWD by sequentially excluding each of the 9 contributing studies. The pooled MDs ranged from 20.95 to 34.44 m, remained positive, and retained 95% CIs that excluded the null in every analysis. The corresponding NNF prediction intervals nevertheless included the null throughout.

Exclusion of Madina et al [47] was accompanied by a decrease in *I*^2^ from 87.8% to 42.5%, while the pooled MD remained statistically significant (MD 20.95, 95% CI 3.57-38.36 m). This finding indicates that the relative inconsistency among study results was lower after exclusion of this study; however, the change in *I*^2^ was not interpreted as an absolute reduction in between-study heterogeneity. Exclusion of the other individual studies did not materially reduce *I*^2^, which ranged from 87.2% to 89.4%; however, these relative inconsistency estimates were interpreted alongside τ^2^and the prediction intervals rather than as absolute measures of heterogeneity. The complete leave-one-out analysis is presented in Figure S14 in Multimedia Appendix 4.

Overall, the sensitivity analyses preserved the direction of the pooled effects for 6MWD, daily steps, and BMI. Knapp-Hartung-adjusted pooled effects remained statistically significant for 6MWD and daily steps after exclusion of the cluster-randomized trial, whereas the BMI estimate included the null. The waist-circumference estimate increased in magnitude but lost precision after exclusion of the cluster-randomized trial. Prediction intervals continued to include the null where estimable.

### Reporting Biases

Potential small-study effects were assessed for peak VO_2_, for which 10 studies were available. Visual inspection of the funnel plot suggested some asymmetry. Egger regression test yielded an intercept of –2.24 (*P*=.10), which did not reach the prespecified 2-sided significance threshold of *P*<.05. Together with the visual asymmetry, this suggested possible small-study effects, although the evidence was inconclusive. This finding should be interpreted cautiously because only 10 studies were available and funnel plot asymmetry may result from between-study heterogeneity, methodological differences, or other factors rather than publication bias alone (Figure 14).

Formal tests for funnel plot asymmetry were not performed for the remaining outcomes because fewer than 10 studies were included in each meta-analysis, resulting in limited statistical power and potentially unreliable assessments.

**Figure 14:**
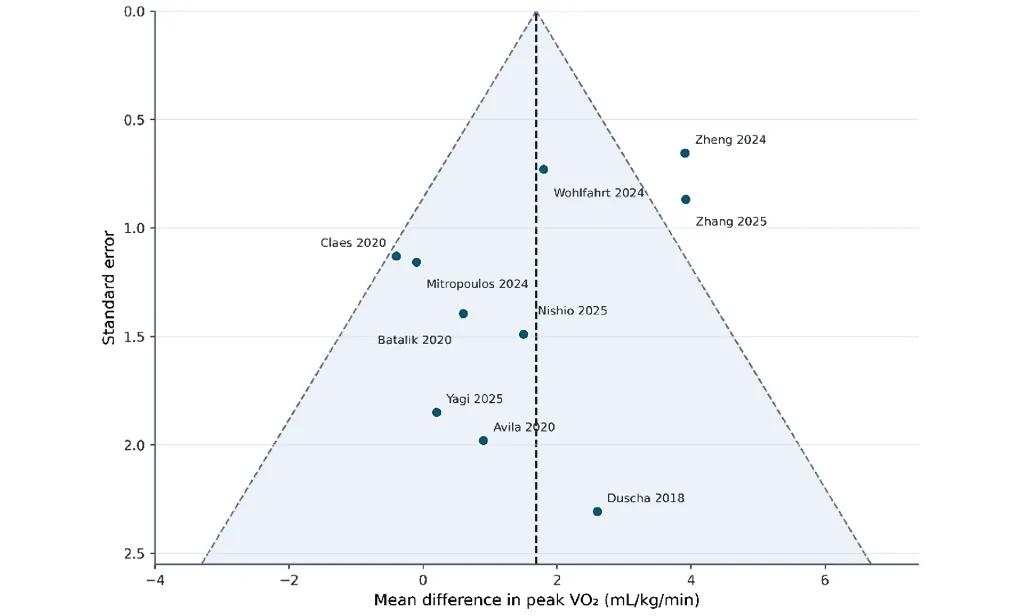
Funnel plot for the meta-analysis of peak oxygen consumption (VO2). The funnel plot shows peak VO2 mean differences against SEs for 10 randomized controlled trials. The vertical line marks the pooled mean difference, and diagonals mark the expected 95% limits. Mild visual asymmetry was not supported by Egger regression (intercept –2.24, SE 1.19; P=.10) and is not direct evidence of publication bias.

### Certainty of Evidence

GRADE certainty ranged from very low to moderate. Peak VO_2_ was moderate, downgraded for inconsistency because effects varied and the NNF 95% prediction interval included the null. 6MWD was moderate, downgraded for inconsistency because effects varied materially and the wide NNF interval included the null. Daily steps were low, downgraded 2 levels for very serious risk of bias, mainly from deviations from intended interventions.

AT and BMI were moderate, each downgraded for imprecision. Waist circumference was low, downgraded for risk of bias and imprecision; only 145 participants contributed, the Knapp-Hartung CI and NNF interval included the null, and cluster-RCT exclusion left 2 studies with less precision. Grip strength was low because of risk of bias and imprecision. QoL was very low because of very serious risk of bias, very serious inconsistency, and imprecision. Table S1 in Multimedia Appendix 4 provides detailed judgments.

## Discussion

### Principal Findings

This systematic review and meta-analysis found favorable average effects of wrist-worn wearable–guided CR delivered through fully remote or hybrid programs on peak VO_2_, 6MWD, and daily steps in patients with CVD, with interventions initiated during phase II or phase III CR. No clear pooled effects were found for AT, BMI, waist circumference, grip strength, or QoL after applying a consistent Knapp-Hartung framework. The prediction intervals for all 3 primary outcomes included the null, indicating uncertainty about whether the favorable average effects would be reproduced in a future comparable setting. Certainty varied across outcomes, and findings for daily steps and QoL were further limited by risk-of-bias concerns. Subgroup and meta-regression findings remain exploratory.

The observed average improvements in peak VO_2_ and 6MWD may be clinically relevant because both outcomes are associated with cardiovascular prognosis and functional independence. Peak VO_2_ is a strong predictor of cardiovascular and all-cause mortality, and modest improvements in cardiorespiratory fitness have been associated with reduced mortality risk [50,51]. The pooled 6MWD estimate was also comparable to commonly reported thresholds for clinically meaningful change in CR populations [52]. These findings are consistent with CR guidelines that identify cardiorespiratory fitness and functional capacity as central treatment targets [3,8]. Nevertheless, the prediction intervals for both outcomes included the null. The pooled estimates should therefore be interpreted as favorable average effects, not effects expected to occur uniformly across future fully remote or hybrid programs.

Subgroup analyses showed numerically larger peak VO_2_ improvements when wrist-worn wearable–guided CR was initiated during phase II rather than phase III and when interventions emphasized aerobic exercise rather than combined aerobic and resistance exercise. However, neither formal interaction test reached statistical significance. These findings should therefore be considered hypothesis-generating rather than evidence that CR phase or exercise type modifies intervention effectiveness. The larger numerical effect during phase II may reflect greater potential for physiological recovery during early outpatient rehabilitation, whereas phase III focuses primarily on the maintenance of previously achieved gains [8,53]. Similarly, aerobic training directly targets the central and peripheral determinants of maximal oxygen uptake [54], although resistance exercise remains important for muscular strength and overall function [55,56]. The small number of studies in some subgroups precludes recommending one CR phase or exercise modality over another.

No statistically significant subgroup differences were identified according to wearable-device type, real-time feedback, or comparator characteristics. Most devices shared core functions such as step counting and HR monitoring, which may have limited the ability to detect device-specific differences [57]. Moreover, the effectiveness of feedback may depend on its frequency, personalization, and integration into exercise prescription rather than simply on whether feedback is available [58]. Control interventions also ranged from usual or minimally supported care to structured center-based, community-based, or wearable-supported programs. Consequently, the absence of statistically significant subgroup interactions should not be interpreted as demonstrating equivalence among devices, feedback approaches, or comparator conditions.

Meta-regression associated higher study-level female proportion with greater peak VO_2_ improvement, while age and intervention duration were not significant moderators. With few studies and aggregate covariates, these exploratory results are susceptible to ecological bias and do not establish greater individual benefit in women or absence of age- or duration-related effects. Remote or hybrid CR may nevertheless improve access for women facing logistical, caregiving, social, or referral barriers [59], a consideration distinct from the observed treatment-effect association.

The pooled analysis indicated a favorable average effect on daily steps with wrist-worn wearable–guided CR; however, confidence is limited because most contributing studies had high overall risk of bias and evidence certainty was low. The prediction interval included the null, indicating that benefit may not be observed consistently across comparable fully remote or hybrid settings. Wearable- and eHealth-supported rehabilitation may facilitate self-monitoring, goal setting, and feedback [58,60]. However, greater step counts do not necessarily indicate reduced sedentary time, higher activity intensity, or sustained long-term behavioral change because these dimensions were not assessed consistently.

The pooled estimates for AT, BMI, and waist circumference were imprecise and included the null after Knapp-Hartung adjustment. AT may respond differently from peak VO_2_ depending on baseline fitness, exercise intensity, and measurement methods [61-63]. The anthropometric point estimates may reflect increased physical activity, as aerobic exercise and sustained activity have been associated with reduced central adiposity [64,65], but most included programs did not provide intensive dietary or weight-management components. For waist circumference, excluding the cluster-randomized trial moved the point estimate farther from the null but reduced precision because only 2 studies remained. These secondary findings should therefore be considered uncertain and should not be attributed specifically to wearable use.

No clear improvement was observed in grip strength, which may reflect the limited emphasis on progressive resistance training in many wrist-worn wearable–guided CR programs. Step-based activity monitoring alone does not provide the training stimulus required to increase muscular strength [55,56]. The available evidence did not establish a clear effect on QoL. The exploratory pooled estimate was highly uncertain because most contributing studies had high overall risk of bias, between-study heterogeneity was extreme, and the certainty of evidence was rated as very low. Therefore, the nonsignificant pooled estimate should not be interpreted as evidence of no effect. Differences in generic and disease-specific instruments and the broader psychosocial and clinical determinants of QoL may also have contributed to the observed variability [66].

Previous reviews of wearable- and eHealth-supported cardiovascular rehabilitation generally found gains in physical activity or exercise capacity [9,10,16-19]. Alam et al [9] reported that quantitative syntheses tended to favor consumer wearables for steps and moderate to vigorous activity, and Heimer et al [10] found that eHealth-supported phase III CR improved activity and capacity with a small QoL benefit. Our QoL estimate was more uncertain, likely reflecting differences in populations, phases, program components, comparators, follow-up, and instruments. Telerehabilitation can extend CR beyond centers [67], but multicomponent benefits cannot be attributed to the wearable alone.

From a clinical perspective, wrist-worn wearable–guided CR may help extend activity monitoring and behavioral support beyond center-based sessions. This may be particularly relevant because conventional CR participation remains limited by travel distance, transportation, program availability, work and caregiving responsibilities, and physical limitations [8,68,69]. Wrist-worn devices have relatively low user burden and can be incorporated into daily life [70,71]. Their most appropriate role may therefore be to complement structured multidisciplinary CR by supporting individualized goals, activity monitoring, and communication between patients and rehabilitation teams rather than to replace supervised exercise prescription or comprehensive secondary prevention.

This review focused on a clinically relevant and scalable category of wearable technology and included only RCTs. Absolute effect measures enhanced clinical interpretability, and prediction intervals distinguished uncertainty surrounding average effects from the expected variation across comparable settings. Subgroup, meta-regression, and sensitivity analyses were used to explore potential sources of heterogeneity, and companion publications were linked to prevent double counting.

Limitations include material variation in effects, especially for 6MWD and QoL, arising from populations, CR phase, duration, prescriptions, wearable functions, comparators, and timing. *I*^2^ was therefore interpreted with τ^2^, prediction intervals, and clinical diversity. Few studies supported several outcomes and subgroups; aggregate meta-regression had limited power and could not establish patient-level modification. Risk-of-bias concerns included deviations, missing data, and outcome measurement. Heterogeneous multicomponent interventions prevented isolating wearable effects, and limited postintervention follow-up precluded conclusions about durability or cardiovascular events. The review lacked prospective registration and a published protocol; some exploratory-analysis eligibility was refined after extraction, and the comparator subgroup was post hoc. Restriction to peer-reviewed English- or Korean-language full texts and exclusion of gray literature may have increased dissemination bias; funnel plots and Egger regression could assess, but not establish, publication bias.

### Conclusions

Wrist-worn wearable–guided CR delivered through fully remote or hybrid programs may improve average exercise capacity and physical activity in patients with CVD during phase II or III CR. Favorable pooled effects were observed for peak VO_2_, 6MWD, and daily steps; however, the prediction intervals included the null, indicating that these benefits may not be consistently reproduced across future settings. No clear effects were observed for AT, BMI, waist circumference, grip strength, or QoL. When integrated into structured multidisciplinary CR, wrist-worn wearables may provide a scalable means of extending activity monitoring, individualized feedback, and behavioral support beyond supervised clinical encounters. Nevertheless, the limited evidence base, risk-of-bias concerns, intervention heterogeneity, and multicomponent nature of the programs warrant cautious interpretation. Larger, rigorous trials should determine which patients and intervention components derive the greatest benefit and how wearable-derived data can support adaptive, personalized CR.

## Appendix

Multimedia Appendix 1 PRISMA 2020 expanded checklist.

Multimedia Appendix 2 Complete search strategies.

Multimedia Appendix 3 Summary of outcome-specific Risk of Bias assessments.

Multimedia Appendix 4 Supplementary figures and tables.

## Abbreviations

6MWD: 6-minute walk distance
AT: anaerobic threshold
CR: cardiac rehabilitation
CVD: cardiovascular disease
GRADE: Grading of Recommendations Assessment, Development and Evaluation
HR: heart rate
MD: mean difference
PCI: percutaneous coronary intervention
PRISMA: Preferred Reporting Items for Systematic Reviews and Meta-Analyses Literature
PRISMA-S: Preferred Reporting Items for Systematic Reviews and Meta-Analyses Literature Search Extension
QoL: quality of life
RCT: randomized controlled trial
RoB 2: Cochrane Risk of Bias 2
SF-36: 36-Item Short Form Health Survey
SMD: standardized mean difference
VO2: oxygen consumption

## References

[1] Chong B, Jayabaskaran J, Jauhari SM, Chan SP, Goh R, Kueh MTW, Li H, Chin YH, Kong G, Anand VV, Wang J, Muthiah M, Jain V, Mehta A, Lim SL, Foo R, Figtree GA, Nicholls SJ, Mamas MA, Januzzi JL, Chew NWS, Richards AM, Chan MY (2025). Global burden of cardiovascular diseases: projections from 2025 to 2050. Eur J Prev Cardiol.

[2] Kazi DS, Elkind MS, Deutsch A, Dowd WN, Heidenreich P, Khavjou O, Mark D, Mussolino ME, Ovbiagele B, Patel SS, Poudel R, Weittenhiller B, Powell-Wiley TM, Joynt Maddox KE, American Heart Association (2024). Forecasting the economic burden of cardiovascular disease and stroke in the United States through 2050: a presidential advisory from the American Heart Association. Circulation.

[3] Bäck M, Wilhelm M, Marcin T, Machado FVC, Sandberg A, Buccheri S, Castro Conde A, Czapla M, De Rosa S, Gibson I, Gombošová A, Korzh O, La Rovere MT, Locati ET, Masip J, Norekval TM, Novo G, Pedretti RFE, Semb AG, Siller-Matula JM, Ski CF, Sündermann SH, Taylor RS, Veltrop RJA, Hansen D, ESC Guidelines Advisory Group (2026). 2026 ESC guidelines on cardiac rehabilitation. Eur J Prev Cardiol.

[4] Dibben GO, Faulkner J, Oldridge N, Rees K, Thompson DR, Zwisler A, Taylor RS (2023). Exercise-based cardiac rehabilitation for coronary heart disease: a meta-analysis. Eur Heart J.

[5] Lawler PR, Filion KB, Eisenberg MJ (2011). Efficacy of exercise-based cardiac rehabilitation post-myocardial infarction: a systematic review and meta-analysis of randomized controlled trials. Am Heart J.

[6] Suaya JA, Shepard DS, Normand ST, Ades PA, Prottas J, Stason WB (2007). Use of cardiac rehabilitation by Medicare beneficiaries after myocardial infarction or coronary bypass surgery. Circulation.

[7] Ritchey MD, Maresh S, McNeely J, Shaffer T, Jackson SL, Keteyian SJ, Brawner CA, Whooley MA, Chang T, Stolp H, Schieb L, Wright J (2020). Tracking cardiac rehabilitation participation and completion among Medicare beneficiaries to inform the efforts of a national initiative. Circ Cardiovasc Qual Outcomes.

[8] Brown TM, Pack QR, Aberegg E, Brewer LC, Ford YR, Forman DE, Gathright EC, Khadanga S, Ozemek C, Thomas RJ, American Heart Association Exercise‚ Cardiac RehabilitationSecondary Prevention Committee of the Council on Clinical Cardiology; Council on CardiovascularStroke Nursing; Council on LifestyleCardiometabolic Health;Council on Quality of CareOutcomes Research (2024). Core components of cardiac rehabilitation programs: 2024 update: a scientific statement from the American Heart Association and the American Association of Cardiovascular and Pulmonary Rehabilitation. Circulation.

[9] Alam S, Zhang M, Harris K, Fletcher LM, Reneker JC (2023). The impact of consumer wearable devices on physical activity and adherence to physical activity in patients with cardiovascular disease: a systematic review of systematic reviews and meta-analyses. Telemed J E Health.

[10] Heimer M, Schmitz S, Teschler M, Schäfer H, Douma E, Habibovic M, Kop WJ, Meyer T, Mooren FC, Schmitz B (2023). eHealth for maintenance cardiovascular rehabilitation: a systematic review and meta-analysis. Eur J Prev Cardiol.

[11] Taylor RS, Dalal HM, McDonagh STJ (2022). The role of cardiac rehabilitation in improving cardiovascular outcomes. Nat Rev Cardiol.

[12] Thomas RJ, Beatty AL, Beckie TM, Brewer LC, Brown TM, Forman DE, Franklin BA, Keteyian SJ, Kitzman DW, Regensteiner JG, Sanderson BK, Whooley MA (2019). Home-based cardiac rehabilitation: a scientific statement from the American Association of Cardiovascular and Pulmonary Rehabilitation, the American Heart Association, and the American College of Cardiology. Circulation.

[13] Rawstorn JC, Gant N, Direito A, Beckmann C, Maddison R (2016). Telehealth exercise-based cardiac rehabilitation: a systematic review and meta-analysis. Heart.

[14] Widmer RJ, Collins NM, Collins CS, West CP, Lerman LO, Lerman A (2015). Digital health interventions for the prevention of cardiovascular disease: a systematic review and meta-analysis. Mayo Clin Proc.

[15] Dunn J, Runge R, Snyder M (2018). Wearables and the medical revolution. Per Med.

[16] Hannan AL, Harders MP, Hing W, Climstein M, Coombes JS, Furness J (2019). Impact of wearable physical activity monitoring devices with exercise prescription or advice in the maintenance phase of cardiac rehabilitation: systematic review and meta-analysis. BMC Sports Sci Med Rehabil.

[17] Antoniou V, Davos CH, Kapreli E, Batalik L, Panagiotakos DB, Pepera G (2022). Effectiveness of home-based cardiac rehabilitation, using wearable sensors, as a multicomponent, cutting-edge intervention: a systematic review and meta-analysis. J Clin Med.

[18] Yu T, Xu H, Sui X, Zhang X, Pang Y, Yu T, Lian X, Zeng T, Wu Y, Leng X, Li F (2023). Effectiveness of eHealth interventions on moderate-to-vigorous intensity physical activity among patients in cardiac rehabilitation: systematic review and meta-analysis. J Med Internet Res.

[19] Maximidou T, Mons U (2025). Benefits of wearable-based cardiac rehabilitation interventions in secondary prevention of coronary artery disease - a systematic review and meta-analysis. Am J Prev Cardiol.

[20] Zhang S, Wang Y, Wu J, Ma C, Meng X (2025). Effectiveness of smartwatch device on adherence to home-based cardiac rehabilitation in patients with coronary heart disease: randomized controlled trial. JMIR Mhealth Uhealth.

[21] Zheng Y, Guo J, Tian Y, Qin S, Liu X (2024). Effect of home-based cardiac telerehabilitation in patients after percutaneous coronary intervention: a randomized controlled trial. Comput Inform Nurs.

[22] Yagi E, Matsumura K, Uchigashima Y, Shiroyama J, Hase M, Nanba T, Yamada N, Funauchi Y, Ueno M, Togi K, Shirotani M, Nakazawa G (2025). Integration of wearables into a cardiac rehabilitation program and its impact on physical activity and exercise capacity in older patients with cardiovascular disease. Adv Rehabil Sci Pract.

[23] Mitropoulos A, Anifanti M, Koukouvou G, Ntovoli A, Alexandris K, Kouidi E (2024). Exploring the effects of real-time online cardiac telerehabilitation using wearable devices compared to gym-based cardiac exercise in people with a recent myocardial infarction: a randomised controlled trial. Front Cardiovasc Med.

[24] Page MJ, McKenzie JE, Bossuyt PM, Boutron I, Hoffmann TC, Mulrow CD, Shamseer L, Tetzlaff JM, Akl EA, Brennan SE, Chou R, Glanville J, Grimshaw JM, Hróbjartsson A, Lalu MM, Li T, Loder EW, Mayo-Wilson E, McDonald S, McGuinness LA, Stewart LA, Thomas J, Tricco AC, Welch VA, Whiting P, Moher D (2021). The PRISMA 2020 statement: an updated guideline for reporting systematic reviews. BMJ.

[25] Rethlefsen ML, Kirtley S, Waffenschmidt S, Ayala AP, Moher D, Page MJ, Koffel JB, PRISMA-S Group (2021). PRISMA-S: an extension to the PRISMA statement for reporting literature searches in systematic reviews. Syst Rev.

[26] Sterne JAC, Savović J, Page MJ, Elbers RG, Blencowe NS, Boutron I, Cates CJ, Cheng H, Corbett MS, Eldridge SM, Emberson JR, Hernán MA, Hopewell S, Hróbjartsson A, Junqueira DR, Jüni P, Kirkham JJ, Lasserson T, Li T, McAleenan A, Reeves BC, Shepperd S, Shrier I, Stewart LA, Tilling K, White IR, Whiting PF, Higgins JPT (2019). RoB 2: a revised tool for assessing risk of bias in randomised trials. BMJ.

[27] Schünemann HJ, Higgins JPT, Vist GE, Glasziou P, Akl EA, Skoetz N (2019). Chapter 14: completing "summary of findings" tables and grading the certainty of the evidence. Cochrane Handbook for Systematic Reviews of Interventions.

[28] R: a language and environment for statistical computing. The R Core Team.

[29] Brini S, Leung TI (2026). Value and credibility of meta-analysis: tutorial on enhancing methodological rigor and AI-powered efficiency. J Med Internet Res.

[30] Higgins JPT, López-López JA (2026). Reflections on the I-squared index for measuring inconsistency in meta-analysis. Res Synth Methods.

[31] Nagashima K, Noma H, Furukawa T (2019). Prediction intervals for random-effects meta-analysis: a confidence distribution approach. Stat Methods Med Res.

[32] GRADE Working Group (2013). GRADE Handbook for Grading Quality of Evidence and Strength of Recommendations.

[33] GRADEpro GDT (2021). GRADEpro Guideline Development Tool Software.

[34] Wohlfahrt P, Jenča D, Melenovský V, Mrázková J, Šramko M, Kotrč M, Želízko M, Adámková V, Lopez-Jimenez F, Piťha J, Kautzner J (2024). Remote, smart device-based cardiac rehabilitation after myocardial infarction: a pilot, randomized cross-over SmartRehab study. Mayo Clin Proc Digit Health.

[35] Lahtio H, Heinonen A, Paajanen T, Sjögren T (2023). The added value of remote technology in cardiac rehabilitation on physical function, anthropometrics, and quality of life: cluster randomized controlled trial. J Med Internet Res.

[36] Beckie TM, Sengupta A, Dey AK, Dutta K, Ji M, Chellappan S (2024). A mobile health behavior change intervention for women with coronary heart disease: a randomized controlled pilot study. J Cardiopulm Rehabil Prev.

[37] Lindman BR, Gillam LD, Coylewright M, Welt FGP, Elmariah S, Smith SA, McKeel DA, Jackson N, Mukerjee K, Cloud H, Hanna N, Purpura J, Ellis H, Martinez V, Selberg AM, Huang S, Harrell FE (2021). Effect of a pragmatic home-based mobile health exercise intervention after transcatheter aortic valve replacement: a randomized pilot trial. Eur Heart J Digit Health.

[38] Nishio R, Dohi T, Yokoyama M, Nakade T, Takahashi N, Chikata Y, Endo H, Nishiyama H, Okai I, Iwata H, Okazaki S, Miyauchi K, Daida H, Minamino T (2025). Wearable devices in remote cardiac rehabilitation with and without weekly online coaching for patients with coronary artery disease: randomized controlled trial. JMIR Mhealth Uhealth.

[39] Claes J, Cornelissen V, McDermott C, Moyna N, Pattyn N, Cornelis N, Gallagher A, McCormack C, Newton H, Gillain A, Budts W, Goetschalckx K, Woods C, Moran K, Buys R (2020). Feasibility, acceptability, and clinical effectiveness of a technology-enabled cardiac rehabilitation platform (Physical Activity Toward Health-I): randomized controlled trial. J Med Internet Res.

[40] Chokshi NP, Adusumalli S, Small DS, Morris A, Feingold J, Ha YP, Lynch MD, Rareshide CA, Hilbert V, Patel MS (2018). Loss-framed financial incentives and personalized goal-setting to increase physical activity among ischemic heart disease patients using wearable devices: the ACTIVE REWARD randomized trial. J Am Heart Assoc.

[41] Batalik L, Dosbaba F, Hartman M, Batalikova K, Spinar J (2020). Benefits and effectiveness of using a wrist heart rate monitor as a telerehabilitation device in cardiac patients: a randomized controlled trial. Medicine (Baltimore).

[42] Avila A, Claes J, Buys R, Azzawi M, Vanhees L, Cornelissen V (2020). Home-based exercise with telemonitoring guidance in patients with coronary artery disease: does it improve long-term physical fitness?. Eur J Prev Cardiol.

[43] Park LG, Elnaggar A, Lee SJ, Merek S, Hoffmann TJ, Von Oppenfeld J, Ignacio N, Whooley MA (2021). Mobile health intervention promoting physical activity in adults post cardiac rehabilitation: pilot randomized controlled trial. JMIR Form Res.

[44] Duscha BD, Piner LW, Patel MP, Craig KP, Brady M, McGarrah RW, Chen C, Kraus WE (2018). Effects of a 12-week mHealth program on peak VO and physical activity patterns after completing cardiac rehabilitation: a randomized controlled trial. Am Heart J.

[45] Hakala S, Kivistö H, Paajanen T, Kankainen A, Anttila M, Heinonen A, Sjögren T (2021). Effectiveness of distance technology in promoting physical activity in cardiovascular disease rehabilitation: cluster randomized controlled trial, a pilot study. JMIR Rehabil Assist Technol.

[46] Dodson JA, Adhikari S, Schoenthaler A, Hochman JS, Sweeney G, George B, Marzo K, Jennings LA, Kovell LC, Vorsanger M, Pena S, Meng Y, Varghese A, Johanek C, Rojas M, McConnell R, Whiteson J, Troxel AB (2025). Rehabilitation at home using mobile health for older adults hospitalized for ischemic heart disease: the RESILIENT Randomized clinical trial. JAMA Netw Open.

[47] Iqbal SMA, Mahgoub I, Du E, Leavitt MA, Asghar W (2021). Advances in healthcare wearable devices. NPJ Flex Electron.

[48] Ntovoli A, Mitropoulos A, Anifanti M, Koukouvou G, Kouidi E, Alexandris K (2025). Can online exercise using wearable devices improve perceived well-being? A study among patients with coronary artery disease. Sensors (Basel).

[49] Brouwers RWM, van der Poort EKJ, Kemps HMC, van den Akker-van Marle ME, Kraal JJ (2021). Cost-effectiveness of cardiac telerehabilitation with relapse prevention for the treatment of patients with coronary artery disease in the Netherlands. JAMA Netw Open.

[50] Ross R, Blair SN, Arena R, Church TS, Després J-P, Franklin BA, Haskell WL, Kaminsky LA, Levine BD, Lavie CJ, Myers J, Niebauer J, Sallis R, Sawada SS, Sui X, Wisløff Ulrik, American Heart Association Physical Activity Committee of the Council on Lifestyle Cardiometabolic Health, Council on Clinical Cardiology, Council on Epidemiology Prevention, Council on Cardiovascular Stroke Nursing, Council on Functional Genomics Translational Biology, Stroke Council (2016). Importance of assessing cardiorespiratory fitness in clinical practice: a case for fitness as a clinical vital sign: a scientific statement from the American Heart Association. Circulation.

[51] Kodama S, Saito K, Tanaka S, Maki M, Yachi Y, Asumi M, Sugawara A, Totsuka K, Shimano H, Ohashi Y, Yamada N, Sone H (2009). Cardiorespiratory fitness as a quantitative predictor of all-cause mortality and cardiovascular events in healthy men and women: a meta-analysis. JAMA.

[52] Gremeaux V, Troisgros O, Benaïm S, Hannequin A, Laurent Y, Casillas J, Benaïm C (2011). Determining the minimal clinically important difference for the six-minute walk test and the 200-meter fast-walk test during cardiac rehabilitation program in coronary artery disease patients after acute coronary syndrome. Arch Phys Med Rehabil.

[53] Ades PA (2001). Cardiac rehabilitation and secondary prevention of coronary heart disease. N Engl J Med.

[54] Montero D, Diaz-Cañestro C, Lundby C (2015). Endurance training and VO2max: role of maximal cardiac output and oxygen extraction. Med Sci Sports Exerc.

[55] Bassett DR, Howley ET (2000). Limiting factors for maximum oxygen uptake and determinants of endurance performance. Med Sci Sports Exerc.

[56] Kirkman DL, Lee D, Carbone S (2022). Resistance exercise for cardiac rehabilitation. Prog Cardiovasc Dis.

[57] Iqbal SMA, Mahgoub I, Du E, Leavitt MA, Asghar W (2021). Advances in healthcare wearable devices. NPJ Flex Electron.

[58] Brickwood KJ, Watson G, O'Brien J, Williams AD (2019). Consumer-based wearable activity trackers increase physical activity participation: systematic review and meta-analysis. JMIR Mhealth Uhealth.

[59] Resurrección DM, Motrico E, Rigabert A, Rubio-Valera M, Conejo-Cerón S, Pastor L, Moreno-Peral P (2017). Barriers for nonparticipation and dropout of women in cardiac rehabilitation programs: a systematic review. J Womens Health (Larchmt).

[60] Jakicic JM, Davis KK, Rogers RJ, King WC, Marcus MD, Helsel D, Rickman AD, Wahed AS, Belle SH (2016). Effect of wearable technology combined with a lifestyle intervention on long-term weight loss: the IDEA randomized clinical trial. JAMA.

[61] Balady GJ, Arena R, Sietsema K, Myers J, Coke L, Fletcher GF, Forman D, Franklin B, Guazzi M, Gulati M, Keteyian SJ, Lavie CJ, Macko R, Mancini D, Milani RV, American Heart Association Exercise‚ Cardiac Rehabilitation‚Prevention Committee of the Council on Clinical Cardiology, Council on EpidemiologyPrevention, Council on Peripheral Vascular Disease, Interdisciplinary Council on Quality of CareOutcomes Research (2010). Clinician's guide to cardiopulmonary exercise testing in adults: a scientific statement from the American Heart Association. Circulation.

[62] Jones AM, Carter H (2000). The effect of endurance training on parameters of aerobic fitness. Sports Med.

[63] Bensimhon DR, Leifer ES, Ellis SJ, Fleg JL, Keteyian SJ, Piña IL, Kitzman DW, McKelvie RS, Kraus WE, Forman DE, Kao AJ, Whellan DJ, O'Connor CM, Russell SD, HF-ACTION Trial Investigators (2008). Reproducibility of peak oxygen uptake and other cardiopulmonary exercise testing parameters in patients with heart failure (from the heart failure and a controlled trial investigating outcomes of exercise traiNing). Am J Cardiol.

[64] Armstrong A, Jungbluth Rodriguez K, Sabag A, Mavros Y, Parker HM, Keating SE, Johnson NA (2022). Effect of aerobic exercise on waist circumference in adults with overweight or obesity: a systematic review and meta-analysis. Obes Rev.

[65] Waller K, Kaprio J, Kujala UM (2008). Associations between long-term physical activity, waist circumference and weight gain: a 30-year longitudinal twin study. Int J Obes (Lond).

[66] Anderson L, Thompson DR, Oldridge N, Zwisler A, Rees K, Martin N, Taylor RS (2016). Exercise-based cardiac rehabilitation for coronary heart disease. Cochrane Database Syst Rev.

[67] Frederix I, Solmi F, Piepoli M, Dendale P (2017). Cardiac telerehabilitation: a novel cost-efficient care delivery strategy that can induce long-term health benefits. Eur J Prev Cardiol.

[68] Turk-Adawi K, Supervia M, Lopez-Jimenez F, Pesah E, Ding R, Britto RR, Bjarnason-Wehrens B, Derman W, Abreu A, Babu AS, Santos CA, Jong SK, Cuenza L, Yeo TJ, Scantlebury D, Andersen K, Gonzalez G, Giga V, Vulic D, Vataman E, Cliff J, Kouidi E, Yagci I, Kim C, Benaim B, Estany ER, Fernandez R, Radi B, Gaita D, Simon A, Chen S, Roxburgh B, Martin JC, Maskhulia L, Burdiat G, Salmon R, Lomelí H, Sadeghi M, Sovova E, Hautala A, Tamuleviciute-Prasciene E, Ambrosetti M, Neubeck L, Asher E, Kemps H, Eysymontt Z, Farsky S, Hayward J, Prescott E, Dawkes S, Santibanez C, Zeballos C, Pavy B, Kiessling A, Sarrafzadegan N, Baer C, Thomas R, Hu D, Grace SL (2019). Cardiac rehabilitation availability and density around the globe. EClinicalMedicine.

[69] Ruano-Ravina A, Pena-Gil C, Abu-Assi E, Raposeiras S, van 't Hof A, Meindersma E, Bossano Prescott EI, González-Juanatey JR (2016). Participation and adherence to cardiac rehabilitation programs. A systematic review. Int J Cardiol.

[70] Schweizer T, Gilgen-Ammann R (2025). Wrist-worn and arm-worn wearables for monitoring heart rate during sedentary and light-to-vigorous physical activities: device validation study. JMIR Cardio.

[71] Müller AM, Wang NX, Yao J, Tan CS, Low ICC, Lim N, Tan J, Tan A, Müller-Riemenschneider F (2019). Heart rate measures from wrist-worn activity trackers in a laboratory and free-living setting: validation study. JMIR Mhealth Uhealth.

