# Supplementary material for: Effectiveness of Wrist-Worn Wearable–Guided Cardiac Rehabilitation Delivered Through Fully Remote or Hybrid Programs in Patients With Cardiovascular Disease: Systematic Review and Meta-Analysis of Randomized Controlled Trials
Source: JMIR Mhealth Uhealth. 2026 Oct 27;14:e101306. doi: 10.2196/101306 (PMC13752593; doi:10.2196/101306)
Supplement: Supplementary file 2 [file mhealth_v14i1e101306_app2.docx]

**Multimedia Appendix 2. Complete Search Strategies**

**Search periods.** The original search covered January 1, 2015, through November 14, 2025, and was executed on November 14, 2025. The updated search covered January 1, 2025, through August 7, 2026, and was executed on August 7, 2026. Because month- or day-level publication-date limits were not consistently available across databases, the updated search began on January 1, 2025. Updated records were deduplicated against the original search records before screening. Across the eight databases, 1,428 records were retrieved in the original search and 552 records were retrieved in the updated search.

**Search-strategy note.** The same database-specific concepts, controlled vocabulary, free-text terms, Boolean operators, and field tags were used for both searches; only the publication-date limit was changed. No automated search translation or text-mining tools were used, and the strategies did not undergo formal PRESS peer review.

| **PubMed** | | | |
| --- | --- | --- | --- |
| *Platform: National Library of Medicine \| Search dates: November 14, 2025 (original); August 7, 2026 (updated) \| Coverage: January 1, 2015–November 14, 2025 (original); January 1, 2025–August 7, 2026 (updated) \| Records retrieved: 133 (original); 79 (updated)* | | | |
| **Set** | **Exact search strategy** | **Original search, n** | **Updated search, n** |
| #1 | Cardiac Surgical Procedures[mh] OR heart surg*[tw] OR cardiac surg*[tw] OR heart operat*[tw] OR percutaneous coronary intervention[mh] OR percutaneous coronary[tw] OR Coronary Artery Bypass[mh] OR Coronary Artery Bypass*[tw] OR Aortocoronary Bypass*[tw] OR CABG[tw] OR valve surg*[tw] OR aortic surg*[tw] OR aorta surg*[tw] OR cardiac valve annuloplasty[mh] OR valv* annuloplast*[tw] OR valv* replac*[tw] OR heart valv*[tw] OR heart transplantation[mh] OR heart transplant*[tw] OR coronary artery disease[mh] OR coronary arter*[tw] OR acute coronary syndrome[mh] OR acute coronary syndrom*[tw] OR Ischemic Heart Disease*[tw] OR Myocardial Ischemia[mh] OR Myocardial Ischemia[tw] OR myocardial infarction[mh] OR myocardi* infarct*[tw] OR heart attack[tw] OR Angina, Unstable[mh] OR unstable angina[tw] OR angina[tw] OR Heart Valve Diseases[mh] OR Aortic Diseases[mh] OR aortic aneurysm*[tw] OR aortic dissection*[tw] | 1,126,760 | 1,158,512 |
| #2 | wrist-wear*[tw] OR wrist-worn[tw] OR wrist wear*[tw] OR Wearable Electronic Devices[mh] OR Wearable Device*[tw] OR smart watch*[tw] OR smartband*[tw] OR activity track*[tw] OR fitness trackers[mh] OR fitness track*[tw] OR smartwatch*[tw] OR wearable*[tw] OR Fitbit[tw] OR Garmin[tw] OR Apple Watch[tw] OR Galaxy Watch[tw] OR Xiaomi Mi Band[tw] OR Huawei Band[tw] OR telerehab*[tw] OR remote activit* monitor*[tw] OR digital health*[tw] OR sports track*[tw] | 77,947 | 92,788 |
| #3 | Cardiac Rehabilitation[mh] OR Rehab*[tw] OR Telerehabilitation[mh] OR telerehab*[tw] OR secondary prevention[mh] OR second* prevent*[tw] OR telehealth*[tw] OR mHealth[tw] OR eHealth[tw] OR telemedicine[tw] OR telemonitor*[tw] OR digital health[tw] OR exercise training[tw] | 577,578 | 612,916 |
| #4 | #2 AND #3 | 30,632 | 39,390 |
| #5 | #1 AND #4 | 447 | 569 |
| #6 | #1 AND #2 | 947 | 1,153 |
| #7 | ((child*[ti] OR adolescen*[ti]) NOT adult*[ti]) OR pediatric*[ti] OR paediatric*[ti] OR pediatric*[ta] OR paediatric*[ta] | 1,447,992 | 1,515,427 |
| #8 | Original search: 2015:2025[dp] Updated search: 2025:2026[dp] | 14,947,948 | 2,932,509 |
| #9 | Controlled Clinical Trials as Topic[mh] OR Controlled Clinical Trial[pt] OR Placebos[mh:noexp] OR ((random*[tw] OR nonrandom*[tw] OR controlled[tw]) AND trial*[tw]) OR RCT[tw] OR RCTs[tw] OR placebo*[tw] OR sham[tw] OR ((single*[tw] OR double*[tw] OR trebl*[tw] OR tripl*[tw]) AND (blind*[tw] OR mask*[tw] OR dumm*[tw])) | 1,520,174 | 1,589,340 |
| **#10** | **(#5 NOT #7) AND #8 AND #9** | **133** | **79** |

| **Embase** | | | |
| --- | --- | --- | --- |
| *Platform: Elsevier \| Search dates: November 14, 2025 (original); August 7, 2026 (updated) \| Coverage: January 1, 2015–November 14, 2025 (original); January 1, 2025–August 7, 2026 (updated) \| Records retrieved: 321 (original); 130 (updated)* | | | |
| **Set** | **Exact search strategy** | **Original search, n** | **Updated search, n** |
| #1 | 'cardiac surgical procedure*':ti,ab,kw OR (((heart OR cardiac OR aortic OR aorta) NEXT/1 surg*):ti,ab,kw) OR 'heart opera*':ti,ab,kw OR 'percutaneous coronary':ti,ab,kw OR 'coronary artery bypass':ti,ab,kw OR 'aortocoronary bypass':ti,ab,kw OR cabg:ti,ab,kw OR 'valve surg*':ti,ab,kw OR 'annuloplast*':ti,ab,kw OR 'val* replac*':ti,ab,kw OR 'heart valv*':ti,ab,kw OR 'heart transplant*':ti,ab,kw OR 'coronary artery':ti,ab,kw OR 'acute coronary syndrom*':ti,ab,kw OR 'ischemic heart disease':ti,ab,kw OR 'heart muscle ischemia':ti,ab,kw OR 'myocardi* infarct*':ti,ab,kw OR 'heart attack':ti,ab,kw OR 'unstable angina pectoris':ti,ab,kw OR 'unstable angina':ti,ab,kw OR 'valvular heart disease':ti,ab,kw OR (((aneurysm OR dissection OR disease) NEXT/1 aort*):ti,ab,kw) | 1,027,909 | 1,069,325 |
| #2 | wrist-wear':ti,ab,kw OR 'wrist wear*':ti,ab,kw OR 'wrist worn':ti,ab,kw OR 'wearable electronic device*':ti,ab,kw OR 'smart watch':ti,ab,kw OR smartband*:ti,ab,kw OR ((activity OR fitness OR sports) NEXT/1 track*):ti,ab,kw OR wearable*:ti,ab,kw OR fitbit:ti,ab,kw OR garmin:ti,ab,kw OR 'apple watch':ti,ab,kw OR 'galaxy watch':ti,ab,kw OR 'xiaomi mi band':ti,ab,kw OR 'huawei band':ti,ab,kw OR telerehab*:ti,ab,kw OR 'remote activity monitor*':ti,ab,kw OR 'digital health':ti,ab,kw | 75,073 | 90,710 |
| #3 | heart rehab*':ti,ab,kw OR 'cardiac rehab*':ti,ab,kw OR telerehab*:ti,ab,kw OR 'second* prevent*':ti,ab,kw OR telehealth*:ti,ab,kw OR mhealth:ti,ab,kw OR ehealth:ti,ab,kw OR telemedi*:ti,ab,kw OR telemonitor*:ti,ab,kw OR 'digital health':ti,ab,kw OR 'exercise train*':ti,ab,kw | 201,770 | 220,906 |
| #4 | #2 AND #3 | 25,311 | 32,652 |
| #5 | #1 AND #4 | 604 | 723 |
| #6 | #1 AND #2 | 1,448 | 1,699 |
| #7 | ((child* OR adolescen*) NOT adult*):ti OR p*ediatric*:ti OR p*ediatric*:jt | 2,231,858 | 2,329,334 |
| #8 | Original search: [2015-2025]/py Updated search: [2025-2026]/py | 19,279,977 | 3,488,756 |
| #9 | ('clinical trial (topic)'/exp OR 'clinical trial'/exp OR [controlled clinical trial]/lim OR [randomized controlled trial]/lim OR randomization/exp OR 'double blind procedure'/de OR 'single blind procedure'/de OR placebo/de OR (random* OR placebo* OR sham OR RCT OR RCTs OR trial* OR CT.gov OR ClinicalTrials.gov OR ICTRP):ti,ab,kw OR ((single* OR double* OR treb* OR tripl*) AND (blind* OR mask* OR dumm*)):ti,ab,kw) | 5,443,058 | 5,778,553 |
| **#10** | **(#5 NOT #7) AND #8 AND #9** | **321** | **130** |

| **Cochrane Central Register of Controlled Trials (CENTRAL)** | | | |
| --- | --- | --- | --- |
| *Platform: Cochrane Library \| Search dates: November 14, 2025 (original); August 7, 2026 (updated) \| Coverage: January 1, 2015–November 14, 2025 (original); January 1, 2025–August 7, 2026 (updated) \| Records retrieved: 566 (original); 184 (updated)* | | | |
| **Set** | **Exact search strategy** | **Original search, n** | **Updated search, n** |
| #1 | [mh "Cardiac Surgical Procedures"] OR [mh "Coronary Artery Bypass"] OR [mh "percutaneous coronary intervention"] OR [mh "cardiac valve annuloplasty"] OR [mh "acute coronary syndrome"] OR [mh "heart transplantation"] OR [mh "coronary artery disease"] OR [mh "Myocardial Ischemia"] OR [mh "myocardial infarction"] OR [mh "Angina, Unstable"] OR [mh "Heart Valve Diseases"] OR [mh "Aortic Diseases"] OR 'cardiac surgical procedure*':ti,ab,kw OR (((heart OR cardiac OR aortic OR aorta) NEXT/1 surg*):ti,ab,kw) OR 'heart opera*':ti,ab,kw OR 'percutaneous coronary':ti,ab,kw OR 'coronary artery bypass':ti,ab,kw OR 'aortocoronary bypass':ti,ab,kw OR cabg:ti,ab,kw OR 'valve surg*':ti,ab,kw OR 'annuloplast*':ti,ab,kw OR 'valv* replac*':ti,ab,kw OR 'heart valv*':ti,ab,kw OR 'heart transplant*':ti,ab,kw OR 'coronary artery disease*':ti,ab,kw OR 'acute coronary syndrom*':ti,ab,kw OR 'ischemic heart disease':ti,ab,kw OR 'heart muscle ischemia':ti,ab,kw OR 'myocardi* infarct*':ti,ab,kw OR 'heart attack':ti,ab,kw OR 'unstable angina pectoris':ti,ab,kw OR 'unstable angina':ti,ab,kw OR 'valvular heart disease':ti,ab,kw OR (((aneurysm OR dissection OR disease) NEXT/1 aort*):ti,ab,kw) | 124,157 | 128,651 |
| #2 | [mh "Wearable Electronic Devices"] OR [mh "fitness trackers"] OR 'wrist-wear':ti,ab,kw OR 'wrist wear*':ti,ab,kw OR 'wrist worn':ti,ab,kw OR 'wearable electronic device*':ti,ab,kw OR 'smart watch':ti,ab,kw OR smartband*:ti,ab,kw OR ((activity OR fitness OR sports) NEXT/1 track*):ti,ab,kw OR wearable*:ti,ab,kw OR fitbit:ti,ab,kw OR garmin:ti,ab,kw OR 'apple watch':ti,ab,kw OR 'galaxy watch':ti,ab,kw OR 'xiaomi mi band':ti,ab,kw OR 'huawei band':ti,ab,kw OR telerehab*:ti,ab,kw OR 'remote activity monitor*':ti,ab,kw OR 'digital health':ti,ab,kw | 19,598 | 23,453 |
| #3 | [mh "Cardiac Rehabilitation"] OR [mh "Telerehabilitation"] OR [mh "secondary prevention"] OR 'heart rehab*':ti,ab,kw OR 'cardiac rehab*':ti,ab,kw OR telerehab*:ti,ab,kw OR 'second* prevent*':ti,ab,kw OR telehealth*:ti,ab,kw OR mhealth:ti,ab,kw OR ehealth:ti,ab,kw OR telemedi*:ti,ab,kw OR telemonitor*:ti,ab,kw OR 'digital health':ti,ab,kw OR 'exercise train*':ti,ab,kw | 170,452 | 185,455 |
| #4 | #2 AND #3 | 14,290 | 17,420 |
| #5 | #1 AND #4 | 705 | 805 |
| #6 | #1 AND #2 | 877 | 997 |
| #7 | ((child* OR adolescen*) NOT adult*):ti OR p*ediatric*:ti OR p*ediatric*:so | 150,360 | 159,099 |
| #8 | Original search: publication years 2015-2025 (interface limit) Updated search: publication years 2025-2026 (interface limit) | 2,328,628 | 2,452,470 |
| #9 | #5 NOT #7 AND #8 | 691 | 785 |
| **#10** | **Original search: #9, limited to publication years 2015-2025 and records indexed as Trials Updated search: #9, limited to publication years 2025-2026 and records indexed as Trials** | **566** | **184** |

| **Web of Science Core Collection** | | | |
| --- | --- | --- | --- |
| *Platform: Clarivate \| Search dates: November 14, 2025 (original); August 7, 2026 (updated) \| Coverage: January 1, 2015–November 14, 2025 (original); January 1, 2025–August 7, 2026 (updated) \| Records retrieved: 143 (original); 74 (updated)* | | | |
| **Set** | **Exact search strategy** | **Original search, n** | **Updated search, n** |
| #1 | TS=("cardiac surgical procedure*" OR "heart surg*" OR "cardiac surg*" OR "heart operat*" OR "percutaneous coronary intervent*" OR "percutaneous coronary*" OR "coronary artery bypass" OR "aortocoronary bypass*" OR CABG OR "valve surg*" OR "aortic surg*" OR "aorta surg*" OR "cardiac valve annuloplasty" OR "valv* annuloplast*" OR "valv* replac*" OR "heart valv*" OR "heart transplant*" OR "coronary arter*" OR "acute coronary syndrome*" OR "ischemic heart disease*" OR "myocardial ischemia*" OR "myocardial infarct*" OR "heart attack" OR "unstable angina" OR angina OR "aortic aneurysm*" OR "aortic dissection*") | 957,925 | 986,568 |
| #2 | TS=("wrist-wear*" OR "wrist-worn" OR "wrist wear*" OR "wearable electronic device*" OR "wearable device*" OR "smart watch*" OR smartband* OR "activity track*" OR "fitness track*" OR smartwatch* OR wearable* OR Fitbit OR Garmin OR "Apple Watch" OR "Galaxy Watch" OR "Xiaomi Mi Band" OR "Huawei Band" OR telerehab* OR "remote activit* monitor*" OR "digital health*" OR "sports track*") | 143,917 | 167,141 |
| #3 | TS=("cardiac rehabilitation" OR rehab* OR telerehab* OR "second* prevent*" OR telehealth* OR mHealth OR eHealth OR telemedicine OR telemonitor* OR "digital health" OR "exercise train*") | 495,770 | 530,450 |
| #4 | #2 AND #3 | 32,641 | 40,589 |
| #5 | #1 AND #4 | 515 | 615 |
| #6 | #1 AND #2 | 1,206 | 1,420 |
| #7 | (TI=(child* OR adolescen* OR pediatric* OR paediatric*) OR SO=("pediatric* OR paediatric*")) NOT TI=(adult*) | 1,674,485 | 1,745,585 |
| #8 | Original search: PY=2015-2025 Updated search: PY=2025-2026 | 34,574,303 | 6,531,471 |
| #9 | TS=("Controlled Clinical Trial" OR ((random* OR nonrandom* OR controlled) NEAR/1 trial*) OR RCT* OR placebo* OR sham OR  ((single* OR double* OR trebl* OR tripl*) NEAR/1 (blind* OR mask* OR dumm*))) | 1,363,599 | 1,428,638 |
| **#10** | **(#5 NOT #7) AND #8 AND #9** | **143** | **74** |

| **Scopus** | | | |
| --- | --- | --- | --- |
| *Platform: Elsevier \| Search dates: November 14, 2025 (original); August 7, 2026 (updated) \| Coverage: January 1, 2015–November 14, 2025 (original); January 1, 2025–August 7, 2026 (updated) \| Records retrieved: 209 (original); 45 (updated)* | | | |
| **Set** | **Exact search strategy** | **Original search, n** | **Updated search, n** |
| #1 | TITLE-ABS-KEY ( "cardiac surgical procedure*" OR "heart surg*" OR "cardiac surg*" OR "heart operat*" OR "percutaneous coronary intervent*" OR "percutaneous coronary*" OR "coronary artery bypass" OR "aortocoronary bypass*" OR CABG OR "valve surg*" OR "aortic surg*" OR "aorta surg*" OR "cardiac valve annuloplasty" OR "valv* annuloplast*" OR "valv* replac*" OR "heart valv*" OR "heart transplant*" OR "coronary arter*" OR "acute coronary syndrome*" OR "ischemic heart disease*" OR "myocardial ischemia*" OR "myocardial infarct*" OR "heart attack" OR "unstable angina" OR angina OR "aortic aneurysm*" OR "aortic dissection*" ) | 1,354,266 | 1,400,847 |
| #2 | TITLE-ABS-KEY ( "wrist-wear*" OR "wrist-worn" OR "wrist wear*" OR "wearable electronic device*" OR "wearable device*" OR "smart watch*" OR smartband* OR "activity track*" OR "fitness track*" OR smartwatch* OR wearable* OR Fitbit OR Garmin OR "Apple Watch" OR "Galaxy Watch" OR "Xiaomi Mi Band" OR "Huawei Band" OR telerehab* OR "remote activit* monitor*" OR "digital health*" OR "sports track*" ) | 187,869 | 222,492 |
| #3 | TITLE-ABS-KEY ( "cardiac rehabilitation" OR rehab* OR telerehab* OR "second* prevent*" OR telehealth* OR mHealth OR eHealth OR telemedicine OR telemonitor* OR "digital health" OR "exercise train*" ) | 762,765 | 824,141 |
| #4 | #2 AND #3 | 44,428 | 57,376 |
| #5 | #1 AND #4 | 835 | 1,036 |
| #6 | #1 AND #2 | 1,818 | 2,187 |
| #7 | TITLE ( child* OR adolescen* ) AND NOT TITLE ( adult* ) ) OR TITLE ( pediatric* OR paediatric* ) | 1,848,417 | 1,934,056 |
| #8 | Original search: PUBYEAR > 2014 AND PUBYEAR < 2026 Updated search: PUBYEAR > 2024 AND PUBYEAR < 2027 | 41,247,326 | 2,923,051 |
| #9 | TITLE-ABS-KEY ( "controlled clinical trial" OR placebo* OR sham OR ( random* W/2 trial* ) OR ( controlled W/2 trial* ) OR ( single* W/2 blind* ) OR ( double* W/2 blind* ) OR ( tripl* W/2 blind* ) OR ( trebl* W/2 blind* ) ) | 1,846,550 | 1,926,506 |
| **#10** | **(#5 NOT #7) AND #8 AND #9** | **209** | **45** |

| **CINAHL** | | | |
| --- | --- | --- | --- |
| *Platform: EBSCOhost \| Search dates: November 14, 2025 (original); August 7, 2026 (updated) \| Coverage: January 1, 2015–November 14, 2025 (original); January 1, 2025–August 7, 2026 (updated) \| Records retrieved: 44 (original); 20 (updated)* | | | |
| **Set** | **Exact search strategy** | **Original search, n** | **Updated search, n** |
| #1 | MH Cardiac Surgery+ OR MH Percutaneous Coronary Intervention OR MH Coronary Arteriosclerosis OR MH Myocardial Ischemia+ OR MH Heart Diseases+ MH Aortic Diseases+ OR TX (((heart OR cardiac OR valv* OR aort*) N2 surg*) OR CABG OR (operat* OR valv* OR transplant* OR disease* OR attack) N2 heart)) | 379,026 | 380,758 |
| #2 | MH Accelerometers+ OR TX ((wrist OR device*) N4 wear*) OR wrist worn OR smart watch* OR smartband* OR telerehab* OR remote activit* monitor* OR digital health) OR XB ( fitbit OR garmin OR apple watch OR galaxy watch OR xiaomi mi band OR huawei band) | 21,301 | 25,111 |
| #3 | MH Rehabilitation, Cardiac+ OR MH Telerehabilitation OR TX ( second* prevent* OR telehealth OR telemedicine OR telemonitor* OR mhealth OR ehealth OR digital health OR exercise train*) | 162,869 | 172,254 |
| #4 | #2 AND #3 | 10,960 | 14,103 |
| #5 | #1 AND #4 | 621 | 714 |
| #6 | #1 AND #2 | 1,241 | — |
| #7 | TI (((child* OR adolescen*) NOT adult*) OR pediatric* OR paediatric*) OR SO (pediatric* OR paediatric*) | 691,026 | 708,322 |
| #8 | Original search: PY 2015-2025 Updated search: PY 2025-2026 | 4,838,210 | 719,958 |
| #9 | MH Randomized Controlled Trials+ OR PT ( Randomized Controlled Trials) TX ((random* OR nonrandom* OR control*) N2 trial*) OR RCT* OR placebo* OR sham OR (single* OR double* OR trebl* OR tripl*) N2 (blind* OR mask* OR dumm*)) | 278,646 | 286,916 |
| **#9** | **(#5 NOT #7) AND #8 AND #9; limited to peer-reviewed publications** | **44** | **20** |

| **KoreaMed** | | | |
| --- | --- | --- | --- |
| *Platform: Korean Association of Medical Journal Editors \| Search dates: November 14, 2025 (original); August 7, 2026 (updated) \| Coverage: January 1, 2015–November 14, 2025 (original); January 1, 2025–August 7, 2026 (updated) \| Records retrieved: 11 (original); 16 (updated)* | | | |
| **Set** | **Exact search strategy** | **Original search, n** | **Updated search, n** |
| #1 | rehab* AND wear* AND heart | 7 | 16 |
| #2 | rehab* AND wear* AND cardiac | 7 | 8 |
| #3 | #1 OR #2 | 11 | 20 |
| #4 | (wear* OR smart) AND cardiac | 10 | 10 |
| #5 | wear* AND (cardiac OR heart) | 27 | 32 |
| #6 | (wear* OR smart) AND (heart disease*) | 14 | 15 |
| #7 | smart* AND (cardiac OR heart) | 27 | 29 |
| **#8** | **#4 OR #5 OR #6 OR #7; original search limited to publication years 2015-2025 and updated search limited to publication years 2025-2026** | **11** | **16** |

| **KMbase** | | | |
| --- | --- | --- | --- |
| *Platform: Korean Medical Database \| Search dates: November 14, 2025 (original); August 7, 2026 (updated) \| Coverage: January 1, 2015–November 14, 2025 (original); January 1, 2025–August 7, 2026 (updated) \| Records retrieved: 1 (original); 4 (updated)* | | | |
| **Set** | **Exact search strategy** | **Original search, n** | **Updated search, n** |
| #1 | TS=("cardiac surgical procedure*" OR "heart surg*" OR "cardiac surg*" OR "heart operat*" OR "percutaneous coronary intervent*" OR "percutaneous coronary*" OR "coronary artery bypass" OR "aortocoronary bypass*" OR CABG OR "valve surg*" OR "aortic surg*" OR "aorta surg*" OR "cardiac valve annuloplasty" OR "valv* annuloplast*" OR "valv* replac*" OR "heart valv*" OR "heart transplant*" OR "coronary arter*" OR "acute coronary syndrome*" OR "ischemic heart disease*" OR "myocardial ischemia*" OR "myocardial infarct*" OR "heart attack" OR "unstable angina" OR angina OR "aortic aneurysm*" OR "aortic dissection*") | 7,463 | 7,639 |
| #2 | TS=("wrist-wear*" OR "wrist-worn" OR "wrist wear*" OR "wearable electronic device*" OR "wearable device*" OR "smart watch*" OR smartband* OR "activity track*" OR "fitness track*" OR smartwatch* OR wearable* OR Fitbit OR Garmin OR "Apple Watch" OR "Galaxy Watch" OR "Xiaomi Mi Band" OR "Huawei Band" OR telerehab* OR "remote activit* monitor*" OR "digital health*" OR "sports track*") | 2,964 | 3,358 |
| #3 | TS=("cardiac rehabilitation" OR rehab* OR telerehab* OR "second* prevent*" OR telehealth* OR mHealth OR eHealth OR telemedicine OR telemonitor* OR "digital health" OR "exercise train*") | 15,734 | 16,606 |
| #4 | #2 AND #3 | 344 | 449 |
| #5 | #1 AND #4 | 1 | 5 |
| #6 | #1 AND #2 | 13 | 18 |
| #7 | (TI=(child* OR adolescen* OR pediatric* OR paediatric*) OR SO=("pediatric* OR paediatric*")) NOT TI=(adult*) | 67,485 | 70,666 |
| **#8** | **#5 NOT #7; no publication-type limit was applied because of the small number of records** | **1** | **4** |
