# Supplementary material for: Effectiveness of Wrist-Worn Wearable–Guided Cardiac Rehabilitation Delivered Through Fully Remote or Hybrid Programs in Patients With Cardiovascular Disease: Systematic Review and Meta-Analysis of Randomized Controlled Trials
Source: JMIR Mhealth Uhealth. 2026 Oct 27;14:e101306. doi: 10.2196/101306 (PMC13752593; doi:10.2196/101306)
Supplement: Supplementary file 3 [file mhealth_v14i1e101306_app3.docx]

**Figure S1.** Risk-of-bias assessment by outcome using the design-appropriate Cochrane Risk of Bias 2 tools. Traffic-light plots show outcome-level RoB 2 judgments using design-appropriate tools and harmonized domains. D1* covers randomization and, for the cluster RCT, recruitment timing; D2† covers deviations and, for the crossover RCT, period and carryover effects; D3-D5 cover missing data, outcome measurement, and selective reporting. Bar plots summarize overall judgments. *For the cluster RCT, D1 includes the additional timing-of-identification or recruitment judgment. †For the crossover RCT, D2 includes period- and carryover-related signaling questions.
