# Supplementary material for: Effectiveness of Wrist-Worn Wearable–Guided Cardiac Rehabilitation Delivered Through Fully Remote or Hybrid Programs in Patients With Cardiovascular Disease: Systematic Review and Meta-Analysis of Randomized Controlled Trials
Source: JMIR Mhealth Uhealth. 2026 Oct 27;14:e101306. doi: 10.2196/101306 (PMC13752593; doi:10.2196/101306)
Supplement: Supplementary file 4 [file mhealth_v14i1e101306_app4.docx]

Figure S1. Detailed forest plot of the subgroup analysis of peak VO₂ according to CR phase at intervention initiation.

RCTs compared wrist-worn wearable-guided CR delivered through fully remote or hybrid programs with control interventions in patients with CVD. Random-effects MDs in peak VO₂ (mL/kg/min) are presented with 95% CIs; subgroup-specific between-study variance was estimated using restricted maximum likelihood and pooled-effect CIs used the Knapp-Hartung adjustment. The interaction test compared interventions initiated during Phase II and Phase III (P for interaction=.081). Positive mean differences favor wearable-guided CR. CI, confidence interval; MD, mean difference; VO₂, oxygen consumption; RCT, randomized controlled trial; CR, cardiac rehabilitation; CVD, cardiovascular disease.

Figure S2. Detailed forest plot of the subgroup analysis of peak VO₂ according to the provision of real-time feedback.

RCTs compared wrist-worn wearable-guided CR delivered through fully remote or hybrid programs with control interventions in patients with CVD. Random-effects MDs in peak VO₂ (mL/kg/min) are presented with 95% CIs; subgroup-specific between-study variance was estimated using restricted maximum likelihood and pooled-effect CIs used the Knapp-Hartung adjustment. The interaction test compared interventions without and with real-time feedback (P for interaction=.753). Positive mean differences favor wearable-guided CR. CI, confidence interval; MD, mean difference; VO₂, oxygen consumption; RCT, randomized controlled trial; CR, cardiac rehabilitation; CVD, cardiovascular disease.

Figure S3. Detailed forest plot of the subgroup analysis of peak VO₂ according to wearable-device type.

RCTs compared wrist-worn wearable-guided CR delivered through fully remote or hybrid programs with control interventions in patients with CVD. Random-effects MDs in peak VO₂ (mL/kg/min) are presented with 95% CIs; subgroup-specific between-study variance was estimated using restricted maximum likelihood and pooled-effect CIs used the Knapp-Hartung adjustment. The interaction test compared smartbands and smartwatches (P for interaction=.992). Positive mean differences favor wearable-guided CR. CI, confidence interval; MD, mean difference; VO₂, oxygen consumption; RCT, randomized controlled trial; CR, cardiac rehabilitation; CVD, cardiovascular disease.

Figure S4. Detailed forest plot of the subgroup analysis of peak VO₂ according to exercise type.

RCTs compared wrist-worn wearable-guided CR delivered through fully remote or hybrid programs with control interventions in patients with CVD. Random-effects MDs in peak VO₂ (mL/kg/min) are presented with 95% CIs; subgroup-specific between-study variance was estimated using restricted maximum likelihood and pooled-effect CIs used the Knapp-Hartung adjustment. The interaction test compared aerobic exercise alone and combined aerobic and resistance exercise (P for interaction=.158). Positive mean differences favor wearable-guided CR. CI, confidence interval; MD, mean difference; VO₂, oxygen consumption; RCT, randomized controlled trial; CR, cardiac rehabilitation; CVD, cardiovascular disease.

Figure S5. Detailed forest plot of the subgroup analysis of peak VO₂ according to comparator characteristics.

RCTs compared wrist-worn wearable-guided CR delivered through fully remote or hybrid programs with control interventions in patients with CVD. Random-effects MDs in peak VO₂ (mL/kg/min) are presented with 95% CIs; subgroup-specific between-study variance was estimated using restricted maximum likelihood and pooled-effect CIs used the Knapp-Hartung adjustment. The interaction test compared minimal or usual-care and active or structured CR comparators (P for interaction=.730). Positive mean differences favor wearable-guided CR. CI, confidence interval; MD, mean difference; VO₂, oxygen consumption; RCT, randomized controlled trial; CR, cardiac rehabilitation; CVD, cardiovascular disease.

Figure S6. Detailed forest plot of the subgroup analysis of 6MWD according to CR phase at intervention initiation.

RCTs compared wrist-worn wearable-guided CR delivered through fully remote or hybrid programs with control interventions in patients with CVD. Random-effects MDs in 6MWD (m) are presented with 95% CIs; subgroup-specific between-study variance was estimated using restricted maximum likelihood and pooled-effect CIs used the Knapp-Hartung adjustment. The interaction test compared interventions initiated during Phase II and Phase III (P for interaction=.351). Positive mean differences favor wearable-guided CR. CI, confidence interval; MD, mean difference; 6MWD, 6-minute walk distance; RCT, randomized controlled trial; CR, cardiac rehabilitation; CVD, cardiovascular disease.

Figure S7. Detailed forest plot of the subgroup analysis of 6MWD according to wearable-device type.

RCTs compared wrist-worn wearable-guided CR delivered through fully remote or hybrid programs with control interventions in patients with CVD. Random-effects MDs in 6MWD (m) are presented with 95% CIs; subgroup-specific between-study variance was estimated using restricted maximum likelihood and pooled-effect CIs used the Knapp-Hartung adjustment. The interaction test compared smartbands and smartwatches (P for interaction=.625). Positive mean differences favor wearable-guided CR. CI, confidence interval; MD, mean difference; 6MWD, 6-minute walk distance; RCT, randomized controlled trial; CR, cardiac rehabilitation; CVD, cardiovascular disease.

Figure S8. Detailed forest plot of the subgroup analysis of 6MWD according to exercise type.

RCTs compared wrist-worn wearable-guided CR delivered through fully remote or hybrid programs with control interventions in patients with CVD. Random-effects MDs in 6MWD (m) are presented with 95% CIs; subgroup-specific between-study variance was estimated using restricted maximum likelihood and pooled-effect CIs used the Knapp-Hartung adjustment. The interaction test compared aerobic exercise alone and combined aerobic and resistance exercise (P for interaction=.547). Positive mean differences favor wearable-guided CR. CI, confidence interval; MD, mean difference; 6MWD, 6-minute walk distance; RCT, randomized controlled trial; CR, cardiac rehabilitation; CVD, cardiovascular disease.

Figure S9. Detailed forest plot of the subgroup analysis of 6MWD according to comparator characteristics.

RCTs compared wrist-worn wearable-guided CR delivered through fully remote or hybrid programs with control interventions in patients with CVD. Random-effects MDs in 6MWD (m) are presented with 95% CIs; subgroup-specific between-study variance was estimated using restricted maximum likelihood and pooled-effect CIs used the Knapp-Hartung adjustment. The interaction test compared minimal or usual-care and active or structured CR comparators (P for interaction=.383). Positive mean differences favor wearable-guided CR. CI, confidence interval; MD, mean difference; 6MWD, 6-minute walk distance; RCT, randomized controlled trial; CR, cardiac rehabilitation; CVD, cardiovascular disease.

Figure S10. Forest plot of the sensitivity analysis of 6MWD after exclusion of the cluster-randomized trial.

After exclusion of the cluster-randomized trial, the DerSimonian-Laird random-effects estimate remained statistically significant with a Knapp-Hartung-adjusted CI (MD 33.95 m, 95% CI 7.60-60.30; P=.019), whereas the NNF 95% prediction interval included the null (-67.52 to 135.99 m). The prediction interval therefore indicates uncertainty about consistent benefit in a future comparable setting. Positive MDs favor wearable-guided CR. CI, confidence interval; MD, mean difference; NNF, Nagashima-Noma-Furukawa; 6MWD, 6-minute walk distance; RCT, randomized controlled trial; CR, cardiac rehabilitation; CVD, cardiovascular disease.

Figure S11. Forest plot of the sensitivity analysis of daily steps after exclusion of the cluster-randomized trial.

After exclusion of the cluster-randomized trial, the DerSimonian-Laird random-effects estimate remained statistically significant with a Knapp-Hartung-adjusted CI (MD 1084.5 steps/day, 95% CI 294.6-1961.1; P=.019), whereas the NNF 95% prediction interval included the null (-704.8 to 3077.3 steps/day). The prediction interval therefore indicates uncertainty about consistent benefit in a future comparable setting. Positive MDs favor wearable-guided CR. CI, confidence interval; MD, mean difference; NNF, Nagashima-Noma-Furukawa; RCT, randomized controlled trial; CR, cardiac rehabilitation; CVD, cardiovascular disease.

Figure S12. Forest plot of the sensitivity analysis of BMI after exclusion of the cluster-randomized trial.

After exclusion of the cluster-randomized trial, the point estimate suggested a larger BMI reduction, but both the Knapp-Hartung-adjusted 95% CI and the NNF 95% prediction interval included the null (MD -1.18 kg/m², 95% CI -2.48 to 0.35; P=.076; 95% PI -3.68 to 1.71). Negative MDs favor wearable-guided CR. CI, confidence interval; MD, mean difference; PI, prediction interval; NNF, Nagashima-Noma-Furukawa; BMI, body mass index; RCT, randomized controlled trial; CR, cardiac rehabilitation; CVD, cardiovascular disease.

Figure S13. Forest plot of the sensitivity analysis of waist circumference after exclusion of the cluster-randomized trial.

After exclusion of the cluster-randomized trial, the estimated reduction increased in magnitude, but precision decreased because only 2 studies remained (MD -3.91 cm, Wald 95% CI -8.27 to 0.44; P=.078). An NNF 95% prediction interval was not reported because the remaining study count was below that for which the method has demonstrated adequate performance. Negative MDs favor wearable-guided CR. CI, confidence interval; MD, mean difference; NNF, Nagashima-Noma-Furukawa; RCT, randomized controlled trial; CR, cardiac rehabilitation; CVD, cardiovascular disease.

Figure S14. Leave-one-out sensitivity analysis of 6MWD.

The analysis sequentially omitted each of the 9 RCTs comparing wrist-worn wearable-guided CR delivered through fully remote or hybrid programs with control interventions in patients with CVD. The full-analysis pooled estimate was MD 31.03 m (Knapp-Hartung-adjusted 95% CI 7.66-53.92; NNF 95% PI -62.11 to 123.18). Across all leave-one-out analyses, the pooled MD remained positive and every Knapp-Hartung-adjusted 95% CI excluded the null, while every NNF 95% prediction interval included the null. Omission of Madina et al. reduced I² from 87.8% to 42.5% while preserving the average effect (MD 20.95 m, 95% CI 3.57-38.36). The solid vertical line indicates the null and the dotted line indicates the full-analysis pooled MD. CI, confidence interval; MD, mean difference; PI, prediction interval; NNF, Nagashima-Noma-Furukawa; 6MWD, 6-minute walk distance; RCT, randomized controlled trial; CR, cardiac rehabilitation; CVD, cardiovascular disease.

Table S1. The detailed GRADE assessments

| Certainty assessment | | | | | | | № of patients | | | Effect | | Certainty | Importance |
| --- | --- | --- | --- | --- | --- | --- | --- | --- | --- | --- | --- | --- | --- |
| № of studies | Study design | Risk of bias | Inconsistency | Indirectness | Imprecision | Other considerations | wearable device | | usual care | Relative (95% CI) | Absolute (95% CI) |  |  |
| Peak VO₂ | | | | | | | | | | | | | |
| 10 | randomized trials | not serious | seriousᵃ | not serious | not serious | none | 298 | 293 | | - | MD 1.69 mL/kg/min higher (0.46 to 2.92 higher) | ⨁⨁⨁◯ Moderateᵃ | CRITICAL |
| 6MWD | | | | | | | | | | | | | |
| 9 | randomized trials | seriousᵇ | seriousᶜ | not serious | not serious | none | 645 | 449 | | - | MD 31.03 m higher (7.66 to 53.92 higher) | ⨁⨁◯◯ Lowᵇ,ᶜ | CRITICAL |
| Daily steps | | | | | | | | | | | | | |
| 8 | randomized trials | very seriousᵈ | not serious | not serious | not serious | none | 238 | | 236 | - | MD 1062.8 steps/day higher (383.5 to 1825.0 higher) | ⨁⨁◯◯ Lowᵈ | CRITICAL |
| Waist circumference | | | | | | | | | | | | | |
| 3 | randomized trials | seriousᵉ | not serious | not serious | seriousᶠ | none | 74 | | 71 | - | MD 2.48 cm lower (5.90 lower to 0.17 higher) | ⨁⨁◯◯ Lowᵉ,ᶠ | IMPORTANT |
| AT | | | | | | | | | | | | | |
| 4 | randomized trials | not serious | not serious | not serious | seriousᵍ | none | 128 | | 128 | - | MD 1.02 mL/kg/min higher (0.22 lower to 2.21 higher) | ⨁⨁⨁◯ Moderateᵍ | IMPORTANT |
| BMI | | | | | | | | | | | | | |
| 5 | randomized trials | not serious | not serious | not serious | seriousʰ | none | 157 | | 157 | - | MD 0.67 kg/m² lower (1.64 lower to 0.24 higher) | ⨁⨁⨁◯ Moderateʰ | IMPORTANT |
| Grip strength | | | | | | | | | | | | | |
| 4 | randomized trials | seriousⁱ | not serious | not serious | seriousʲ | none | 115 | | 121 | - | MD 0.06 kg lower (1.24 lower to 1.08 higher) | ⨁⨁◯◯ Lowⁱ,ʲ | IMPORTANT |
| QoL | | | | | | | | | | | | | |
| 5 | randomized trials | very seriousᵏ | very seriousˡ | not serious | seriousᵐ | none | 290 | | 290 | - | SMD 0.42 SD higher (0.49 lower to 1.34 higher) | ⨁◯◯◯ Very lowᵏ,ˡ,ᵐ | CRITICAL |

CI: confidence interval; MD: mean difference; SMD: standardized mean difference

#### Explanations

a. Downgraded one level for inconsistency because study effects varied materially (I² = 61.6%; τ² = 1.97), and the NNF 95% prediction interval included the null.

b. Downgraded one level for risk of bias because 1 study was judged at high overall risk of bias and the remaining 8 studies had some concerns.

c. Downgraded one level for inconsistency because study effects varied materially (I² = 87.8%; τ² = 1241.17), and the NNF 95% prediction interval was wide and included both no effect and potentially important effects in either direction (−62.11 to 123.18 m).

d. Downgraded two levels for risk of bias because 6 of the 8 included studies were judged at high overall risk of bias, primarily because of deviations from intended interventions.

e. Downgraded one level for risk of bias because all 3 contributing studies had some concerns.

f. Downgraded one level for imprecision because only 145 participants contributed and the Knapp–Hartung-adjusted 95% CI included both a potentially important reduction and no effect. The NNF 95% prediction interval also included the null, and exclusion of the cluster-randomized trial left only 2 studies and produced a less precise estimate.

g. Downgraded one level for imprecision because only 256 participants contributed and the Knapp–Hartung-adjusted 95% CI included both no effect and potentially important benefit.

h. Downgraded one level for imprecision because only 314 participants contributed and the Knapp–Hartung-adjusted 95% CI included both a potentially important reduction and no effect.

i. Downgraded one level for risk of bias because 3 studies had some concerns and 1 study had high overall risk of bias.

j. Downgraded one level for imprecision because only 236 participants contributed and the Knapp–Hartung-adjusted 95% CI included potentially meaningful benefit and harm.

k. Downgraded two levels for risk of bias because 4 of the 5 included studies were judged at high overall risk of bias and the remaining study had some concerns.

l. Downgraded two levels for inconsistency because study effects varied materially (I² = 95.0%; τ² = 0.85), and the included instruments and outcome domains differed clinically.

m. Downgraded one level for imprecision because the Knapp–Hartung-adjusted 95% CI was wide and included substantial effects in either direction.
